# Dynamic and Static Nature of Br_4_*σ*(4c–6e) and Se_2_Br_5_*σ*(7c–10e) in the Selenanthrene System and Related Species Elucidated by QTAIM Dual Functional Analysis with QC Calculations

**DOI:** 10.1155/2020/2901439

**Published:** 2020-07-24

**Authors:** Satoko Hayashi, Taro Nishide, Waro Nakanishi

**Affiliations:** Faculty of Systems Engineering, Wakayama University, 930 Sakaedani, Wakayama 640-8510, Japan

## Abstract

The nature of Br_4_*σ*(4c–6e) of the ^B^Br-∗-^A^Br-∗-^A^Br-∗-^B^Br form is elucidated for SeC_12_H_8_(Br)Se**Br---Br-Br---Br**Se(Br)C_12_H_8_Se, the selenanthrene system, and the models with QTAIM dual functional analysis (QTAIM-DFA). Asterisks (∗) are employed to emphasize the existence of bond critical points on the interactions in question. Data from the fully optimized structure correspond to the static nature of interactions. In our treatment, data from the perturbed structures, around the fully optimized structure, are employed for the analysis, in addition to those from the fully optimized one, which represent the dynamic nature of interactions. The ^A^Br-∗-^A^Br and ^A^Br-∗-^B^Br interactions are predicted to have the CT-TBP (trigonal bipyramidal adduct formation through charge transfer) nature and the typical hydrogen bond nature, respectively. The nature of Se_2_Br_5_*σ*(7c–10e) is also clarified typically, employing an anionic model of [**Br-Se**(C_4_H_4_Se)**-Br---Br---Br-Se**(C_4_H_4_Se)**-Br**]^−^, the 1,4-diselenin system, rather than (BrSeC_12_H_8_)**Br---Se---Br**-**Br---Br-Se**(C_12_H_8_Se)**-Br**, the selenanthrene system.

## 1. Introduction

We have been much interested in the behavior of the linear interactions of the *σ*-type, higher than *σ*(3c–4e: three center-four electron interactions) [1–6], constructed by the atoms of heavier main group elements. We proposed to call such linear interactions the extended hypervalent interactions, *σ*(*m*c–*n*e: 4 ≤ *m*; *m* < *n* < 2*m*), after the hypervalent *σ*(3c–4e). The linear alignments of four chalcogen atoms were first demonstrated in the naphthalene system, bis[8-(phenylchalcogenyl)naphthyl]-1,1′-dichalcogenides [**I**: 1-(8-Ph^B^EC_10_H_6_)^A^E-^A^E(C_10_H_6_^B^EPh-8′)-1′ (^A^E, ^B^E = S and Se)] [7–12]. It was achieved through the preparation and the structural determination by the X-ray crystallographic analysis. The linear ^B^E---^A^E-^A^E---^B^E interactions in **I** are proposed to be analysed as the ^A^E_2_^B^E_2_*σ*(4c–6e) model not by the double ^A^E^B^E_2_*σ*(3c–4e) model. ^A^E_2_^B^E_2_*σ*(4c–6e) in **I** is characterized by the CT interaction of the *n*_p_(^B^E) ⟶ *σ*∗(^A^E–^A^E)←*n*_p_(^B^E) form [8, 10–12], where *n*_p_(^B^E) stands for the p-type nonbonding orbitals of ^B^E and *σ*∗(^A^E-^A^E) are the *σ*∗ orbitals of ^A^E-^A^E. The novel reactivity of ^A^E_2_^B^E_2_*σ*(4c–6e) in **I** was also clarified [8].

*σ*(4c–6e) is the first member of *σ*(*m*c–*n*e: 4 ≤ *m*; *m* < *n* < 2*m*) [7–13]. The *σ*(4c–6e) interactions are strongly suggested to play an important role in the development of high functionalities in materials and in the key processes of biological and pharmaceutical activities, recently. The bonding is applied to a wide variety of fields, such as crystal engineering, supramolecular soft matters, and nanosciences [4, 14–23]. The nature of ^B^E---^A^E and ^A^E-^A^E in ^B^E---^A^E-^A^E---^B^E of ^A^E_2_^B^E_2_*σ*(4c–6e) has been elucidated [24–27] using the quantum theory of atoms in molecules (QTAIM) approach, introduced by Bader [28–37]. The linear interactions of the *σ*(4c–6e) type will form if ^B^E in ^A^E_2_^B^E_2_ is replaced by *X*, giving E_2_X_2_*σ*(4c–6e). The nature of E_2_X_2_*σ*(4c–6e) in the naphthalene system of 1-(8-XC_10_H_6_)E-E(C_10_H_6_X-8′)-1′ [**II** (E, *X*) = (S, Cl), (S, Br), (Se, Cl), and (Se, Br)] was similarly clarified very recently [38].

**Figure 1:**
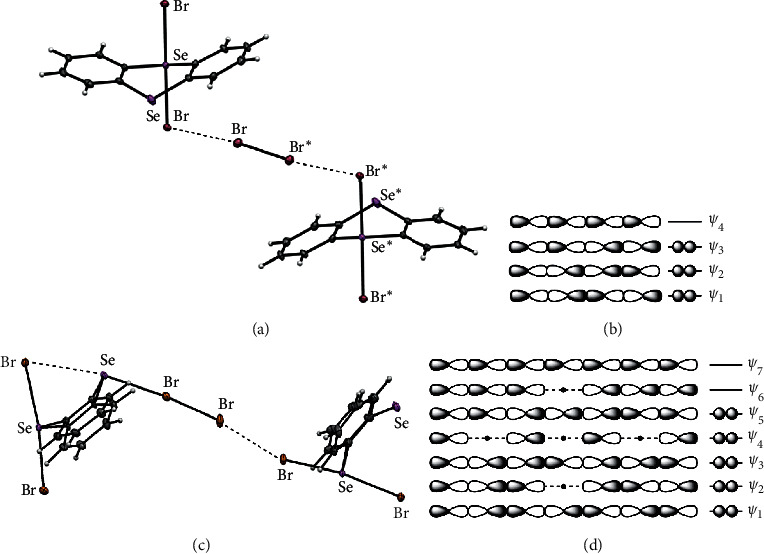
Structure of **1** determined by the X-ray crystallographic analysis (a) and the approximate MO model for *σ*(4c–6e) (b); structure of **2** (c) and the approximate MO model for *σ*(7c–10e) (d).

The *σ*(4c–6e) interaction will also be produced even if both ^B^E and ^A^E in ^A^E_2_^B^E_2_ are replaced by *X*. X_4_*σ*(4c–6e) should also be stabilized through CT of the *n*_p_(*X*) ⟶ *σ*∗(X-X) ← *n*_p_(*X*) form. The energy lowering of the system through the CT interaction must be the driving force for the formation of X_4_*σ*(4c–6e). X_4_*σ*(4c–6e) is the typical kind of halogen bonds, together with E_2_X_2_*σ*(4c–6e), which are of current and continuous interest [39]. Br_4_*σ*(4c–6e) has been clearly established in the selenanthrene system, SeC_12_H_8_(Br)Se**Br**---**Br**-**Br**---**Br**Se(Br)C_12_H_8_Se (**1**), through the preparation and the structural determination by the X-ray crystallographic analysis [39]. The atoms taking part in the linear interaction in question are shown in bold. The structure of (BrSeC_12_H_8_)**Br**---**Se**---**Br**-**Br**---**Br-Se**(C_12_H_8_Se)**-Br** (**2**) was also reported, in addition to **1**, which is suggested to contain Se_2_Br_5_*σ*(7c–10e) since the seven atoms of Se_2_Br_5_ align almost linearly in crystals.  Figure 1 shows the structures of **1** and **2** determined by the X-ray analysis and the approximate MO model for *σ*(4c–6e) and *σ*(7c–10e).

**Figure 2:**
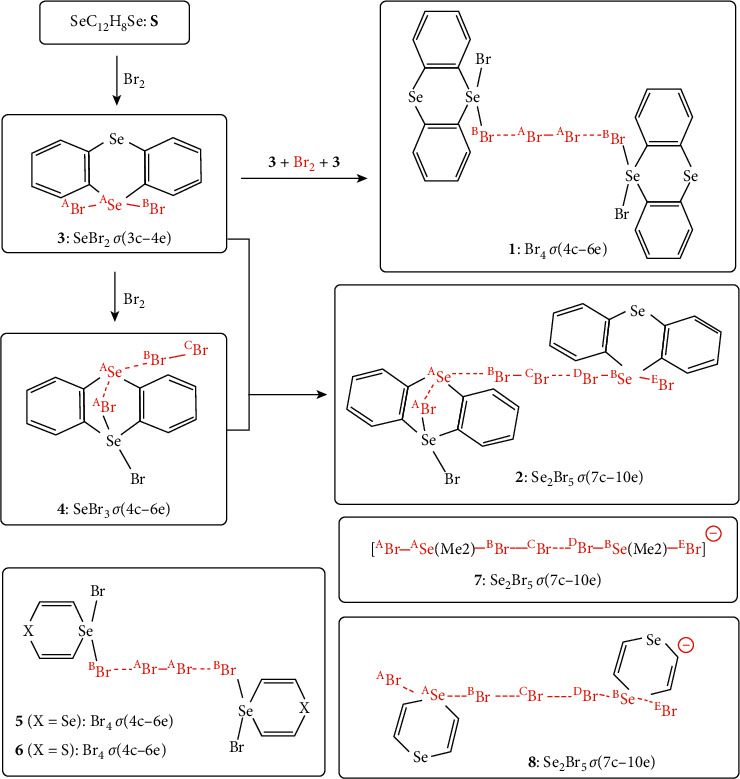
Process assumed for the formation of Br_4_*σ*(4c–6e) in **1** from Se(C_12_H_8_)Se (**S**) *via ***3** and Se_2_Br_5_*σ*(7c–10e) in **2 ***via ***3** and **4**. **5** and **6** with Br_4_*σ*(4c–6e), models of **1**, and **7** and **8** with Se_2_Br_5_*σ*(7c–10e), models of **2**, are also shown. Atoms taking part in the linear interactions are shown by red.

It is challenging to elucidate the nature of Br_4_*σ*(4c–6e) of the *n*_p_(Br) ⟶ *σ*∗(Br-Br)←*n*_p_(Br) form in **1** and Se_2_Br_5_*σ*(7c–10e) in **2**, together with the related species.  Figure 2 illustrates the process assumed for the formation of **1** and **2** from selenanthrene (**S**: SeC_12_H_8_Se). In this process, (SeC_12_H_8_)**Br**-**Se**-**Br** (**3**) should be formed first in the reaction of **S** with **Br**_2_, and then **3** reacts with **Br**_2_ to yield **Br**[Se(Br) (C_12_H_8_)]**Se**---**Br**-**Br** (**4**). The almost linear alignment of **Br**---**Se**---**Br**-**Br** in **4** could be analysed by the SeBr_3_*σ*(4c–6e) model, where the **Br** and **Se** atoms in **4** are placed in close proximity in space. While **1** containing Br_4_*σ*(4c–6e) forms in the reaction of (**3** + Br_2_ + **3**), the reaction of **3** + **4** yields **2**, consisting Se_2_Br_5_*σ*(7c–10e). Both **1** and **2** are recognized as the Br_2_-included species. While XC_4_H_4_(Br)Se**Br**---**Br**-**Br**---**Br**Se(Br)C_4_H_4_X (**5** (*X* = Se) and **6** (*X* = S)), models of **1**, also consisted of Br_4_*σ*(4c–6e), Se_2_Br_5_*σ*(7c–10e) will appear typically in the anionic species, [**Br-Se**(Me_2_)**-Br**---**Br**---**Br**-**Se**(Me_2_)-**Br**]^−^ (**7**) and [**Br-Se**(SeC_4_H_4_)**-Br**---**Br**---**Br**-**Se**(C_4_H_4_Se)**-Br**]^−^ (**8**), models of **2**. Species, **5**, **6**, **7**, and **8**, are shown in Figure 2, where **5**, **6**, and **8** belong to the 1,4-diselenin system.

**Figure 3:**
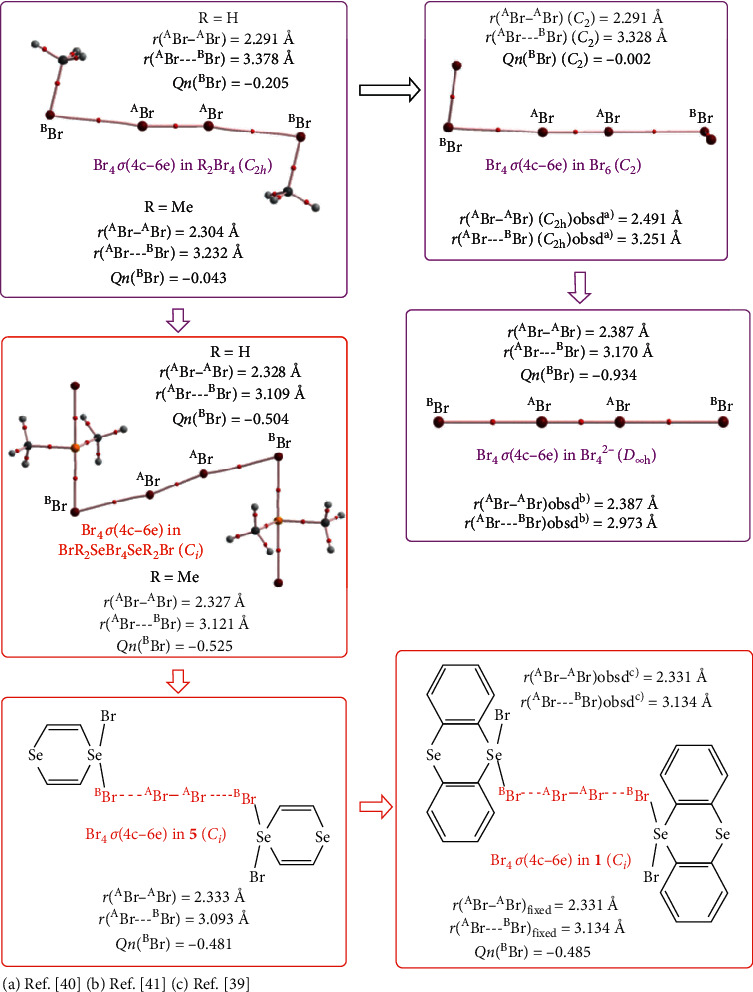
Sequence in the stabilization of Br_4_*σ*(4c–6e), starting from those in G(**A**) to **1** via those of G(**B**).

What are the differences and similarities between X_4_*σ*(4c–6e), E_4_*σ*(4c–6e), and E_2_X_2_*σ*(4c–6e)? The nature of X_4_*σ*(4c–6e) in **1** (*X* = Br) is to be elucidated together with the models. Models, other than **5** and **6**, are also devised to examine the stabilization sequence of Br_4_*σ*(4c–6e). H_2_Br_4_ (*C*_2h_) and Me_2_Br_4_ (*C*_2h_) have the form of R-**Br**---**Br**-**Br**---**Br**-R (RBr_4_R: R = H and Me), which are called the model group **A** (G(**A**)). The electronic efficiency to stabilize Br_4_*σ*(4c–6e) seems small for R in G(**A**). Br_6_ (*C*_2h_) is detected as the partial structure in the crystals of Br_2_ [40]. Br_6_ (*C*_2h_) in the crystals is denoted by Br_6_ (*C*_2h_)_obsd_. The optimized structure of Br_6_ (*C*_2h_) has one imaginary frequency, which belongs to G(**A**), together with Br_6_ (*C*_2h_)_obsd_. The optimized structure of Br_6_ retains the *C*_2_ symmetry, (Br_6_ (*C*_2_)), which also belongs to G(**A**). The CT interaction of the *n*_p_(^B^Br) ⟶ *σ*∗(^A^Br-^A^Br) ⟵ *n*_p_(^B^Br) form in Br_4_*σ*(4c–6e) will be much stabilized if the large negative charge is developed at the ^B^Br atoms in Br-(R_2_)Se-^B^**Br**---^A^**Br**-^A^**Br**---^B^**Br**-Se(R_2_)-Br, where the ∠Se^B^**Br**^A^**Br** is around 90°. The highly negatively charged ^B^**Br** in Br-Se(R_2_)-^B^**Br** (R = H and Me) of *σ*(3c–4e) is employed to stabilize Br_4_*σ*(4c–6e), in this case. The models form G(**B**). The nature of Br_4_*σ*(4c–6e) in **5** and **6** is similarly analysed, which belongs to G(**B**). Br_4_^2−^ (*D*_∞h_) also belongs to G(**B**) although one imaginary frequency was predicted for Br_4_^2−^, if optimized at the MP2 level.  Figure 3 illustrates the story for the stabilization of Br_4_*σ*(4c–6e) in the sequence of the species, starting from G(**A**) to **1**, *via* G(**B**).  Figure 3 also shows the ^A^Br-^A^Br and ^A^Br---^B^Br distances (*r*(^A^Br-^A^Br) and *r*(^A^Br-^B^Br), respectively), together with the charge developed at ^B^Br in the original species of R-^B^Br (*Qn* (^B^Br)), which construct R-^B^Br---^A^Br-^A^Br---^B^Br-R.

A chemical bond or interaction between atoms A and B is denoted by A-B, which corresponds to a bond path (BP) in the quantum theory of atoms in molecules (QTAIM) approach, introduced by Bader [28–37]. We will use A-∗-B for BP, where the asterisk emphasizes the existence of a bond critical point (BCP, ∗) in A-B [28, 29]. (Dots are usually employed to show BCPs in molecular graphs. Therefore, A-•-B would be more suitable to describe the BP with a BCP. Nevertheless, A-∗-B is employed to emphasize the existence of a BCP on the BP in question in our case. BCP is a point along BP at the interatomic surface, where *ρ*(**r**) (charge density) reaches a minimum along the interatomic (bond) path, while it is a maximum on the interatomic surface separating the atomic basins). The chemical bonds and interactions are usually classified by the signs of Laplacian rho (∇^2^*ρ*_b_(**r**_c_)) and *H*_b_(**r**_c_) at BCPs, where *ρ*_b_(**r**_c_) and *H*_b_(**r**_c_) are the charge densities and total electron energy densities at BCPs, respectively (see Scheme S1 in Supplementary File). The relations between *H*_b_(**r**_c_), ∇^2^*ρ*_b_(**r**_c_), *G*_b_(**r**_c_) (the kinetic energy densities), and *V*_b_(**r**_c_) (the potential energy densities) are represented in equations (1) and (2):

(1) $$\begin{array}{c} H_{\text{b}}\left(r_{\text{c}}\right)=G_{\text{b}}\left(r_{\text{c}}\right)+V_{\text{b}}\left(r_{\text{c}}\right), \end{array}$$

(2) $$\begin{array}{c} \left(\frac{\hbar^{2}}{8m}\right)\nabla^{2}\rho_{\text{b}}\left(r_{\text{c}}\right)=H_{\text{b}}\left(r_{\text{c}}\right)-\frac{V_{\text{b}}\left(r_{\text{c}}\right)}{2} \end{array}$$

**Figure 4:**
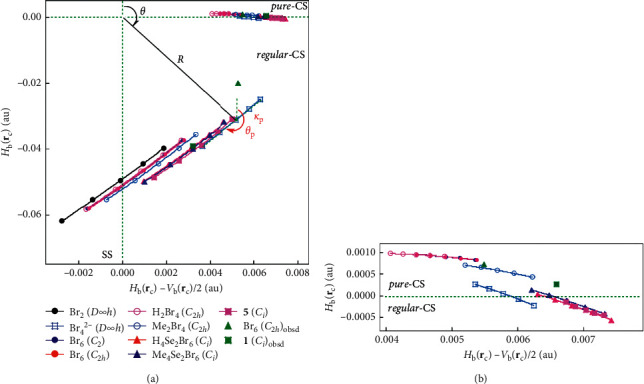
QTAIM-DFA plots of *H*_b_(**r**_c_) versus *H*_b_(**r**_c_) − *V*_b_(**r**_c_)/2 for ^A^Br-∗-^A^Br (a) and ^A^Br-∗-^B^Br (b) in Br_4_*σ*(4c–6e) of the species in Table 1, together with those of the perturbed structures generated with CIV. Marks and colours for the species are shown in the figure.

How can the nature of Br_4_*σ*(4c–6e) and Se_2_Br_5_*σ*(7c–10e) be clarified? For the characterization of interactions in more detail, we recently proposed QTAIM dual functional analysis (QTAIM-DFA) [42–47] for experimental chemists to analyze their own chemical bonds and interaction results based on their own expectations, according to the QTAIM approach [28–37]. *H*_b_(**r**_c_) is plotted versus *H*_b_(**r**_c_) − *V*_b_(**r**_c_)/2 (= (*ћ*^2^/8*m*)∇^2^*ρ*_b_(**r**_c_)) at BCPs in QTAIM-DFA. The classification of interactions by the signs of ∇^2^*ρ*_b_(**r**_c_) and *H*_b_(**r**_c_) is incorporated in QTAIM-DFA. Data from the fully optimized structures correspond to the static natures of the interactions, which are analysed using the polar coordinate (*R*, *θ*), representation [42, 44–46]. Each interaction plot, containing data from both the perturbed structures and the fully optimized one include a specific curve that provides important information about the interaction. This plot is expressed by (*θ*_p_, *κ*_p_), where *θ*_p_ corresponds to the tangent line of the plot and *κ*_p_ is the curvature. The concept of the dynamic nature of interactions has been proposed based on (*θ*_p_, *κ*_p_) [42, 44]. *θ* and *θ*_p_ are measured from the *y*-axis and the *y*-direction, respectively. We call (*R*, *θ*) and (*θ*_p_, *κ*_p_) QTAIM-DFA parameters, which are drawn in Figure 4, exemplified by Br_4_^2−^ (*D*_∞h_). While (*R*, *θ*) classifies the interactions, (*θ*_p_, *κ*_p_) characterizes them.

We proposed a highly reliable method to generate the perturbed structures for QTAIM-DFA very recently [48]. The method is called CIV, which employs the coordinates derived from the compliance force constants *C*_*ij*_ for the internal vibrations. Compliance force constants *C*_*ij*_ are defined as the partial second derivatives of the potential energy due to an external force, as shown in equation (3), where *i* and *j* refer to the internal coordinates and the force constants *f*_*i*_ and *f*_*j*_ correspond to *i* and *j*, respectively. The *C*_*ij*_ values and the coordinates corresponding to the values can be calculated using the compliance 3.0.2 program, released by Brandhorst and Grunenberg [49–52]. The dynamic nature of interactions based on the perturbed structures with CIV is described as the “intrinsic dynamic nature of interactions” since the coordinates are invariant to the choice of the coordinate system:

(3) $$\begin{array}{c} C_{ij}=\frac{\partial^{2}E}{\partial f_{i}\partial f_{j}}. \end{array}$$

QTAIM-DFA has excellent potential for evaluating, classifying, characterizing, and understanding weak to strong interactions according to a unified form. The superiority of QTAIM-DFA to elucidate the nature of interactions, employing the perturbed structures generated with CIV, is explained in the previous papers [48, 53] (see also Figure S2 and Table S2 in Supplementary File). QTAIM-DFA is applied to standard interactions and rough criteria that distinguish the interaction in question from others which are obtained. QTAIM-DFA and the criteria are explained in Supplementary File using Schemes S1–S3, Figures S1 and S2, Table S1, and equations (S1)–(S7). The basic concept of the QTAIM approach is also explained.

We consider QTAIM-DFA, employing the perturbed structures generated with CIV, to be well suited to elucidate the nature of Br_4_*σ*(4c–6e) in **1**, Se_2_Br_5_*σ*(7c–10e) in **2**, and the models derived from **1** and **2**, together with the related linear interactions. The interactions in Br_4_*σ*(4c–6e) are denoted by ^B^Br-∗-^A^Br-∗-^A^Br-∗-^B^Br, where the asterisk emphasizes the existence of a BCP in the interactions, so are those in Se_2_Br_5_*σ*(7c–10e). Herein, we present the results of the investigations on the extended hypervalent interactions in the species, together with the structural feature. Each interaction is classified and characterized, employing the criteria as a reference.

## 2. Methodological Details in Calculations

Calculations were performed employing the Gaussian 09 programs package [54]. The basis sets employed for the calculations were obtained, as implemented from Sapporo Basis Set Factory [55]. The basis sets of the (621/31/2), (6321/621/3), (74321/7421/72), and (743211/74111/721/2+1s1p) forms were employed for C, S, Se, and Br, respectively, with the (31/3) form for H. The basis set system is called BSS-A. All species were calculated employing BSS-A, and the Møller–Plesset second-order energy correlation (MP2) level [56–58] was applied for the optimizations. Optimized structures were confirmed by the frequency analysis. The results of the frequency analysis were used to calculate the *C*_*ij*_ values and the coordinates (**C**_*i*_) corresponding to the values. The DFT level of CAM-B3LYP [59] was also applied when necessary. The QTAIM functions were analysed with the AIM2000 [60] and AIMAll [61] programs.

The method to generate perturbed structures with CIV is the same as that explained in the previous papers [48, 53]. As shown in equation (4), the *i*-th perturbed structure in question (**S**_*iw*_) is generated by the addition of the *i*-th coordinates (**C**_*i*_), derived from *C*_*ij*_, to the standard orientation of a fully optimized structure (**S**_o_) in the matrix representation. The coefficient *f*_*iw*_ in equation (4) controls the structural difference between **S**_*iw*_ and **S**_o_: *f*_*iw*_ is determined to satisfy equation (5) for *r*, where *r* and *r*_o_ stand for the interaction distances in question in the perturbed and fully optimized structures, respectively, with *a*_o_ = 0.52918 Å (Bohr radius). The **C**_*i*_ values of five digits are used to predict **S**_*iw*_:

(4) $$\begin{array}{c} S_{iw}=S_{\text{o}}+f_{iw}\cdot C_{i}, \end{array}$$

(5) $$\begin{array}{c} r=r_{o}+wa_{o}, \end{array}$$

(6) $$\begin{array}{c} y=c_{o}+c_{1}x+c_{2}x^{2}+c_{3}x^{3},\\ \left(R_{c}^{2}:\text{square of correlation coefficient}\right). \end{array}$$

In QTAIM-DFA, *H*_b_(**r**_c_) is plotted versus *H*_b_(**r**_c_) − *V*_b_(**r**_c_)/2 for data of *w* = 0, ±0.05, and ±0.10 in equation (5). Each plot is analysed using a regression curve of the cubic function, as shown in equation (6), where (*x*, *y*) = (*H*_b_(**r**_c_) − *V*_b_(**r**_c_)/2 and *H*_b_(**r**_c_)) (*R*_*c*_^2^ (square of correlation coefficient) > 0.99999 in usual) [46].

## 3. Results and Discussion

### 3.1. Structural Optimizations

**Figure 5:**
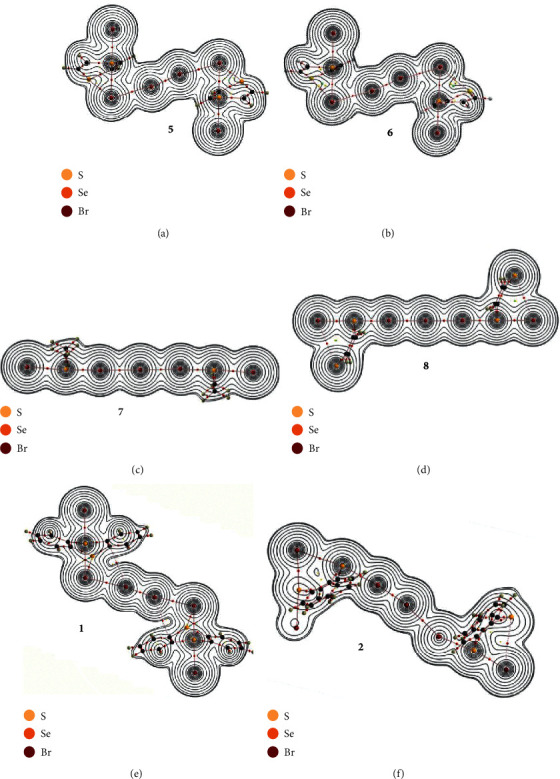
Molecular graphs of **5** (*C*_*i*_) (a), **6** (*C*_*i*_) (b), **7** (*C*_2h_) (c), and **8** (*C*_2h_) (d) drawn on the structures optimized at the MP2 level, together with **1** (*C*_*i*_)_obsd_ (e) and **2** (*C*_1_)_obsd_ (f). Contour plots of *ρ*(**r**) are also drawn on the planes containing the linear interactions. BCPs are denoted by red dots, RCPs (ring critical points) by yellow dots, and CCPs (cage critical points) by green dots. BPs (bond paths) are drawn as pink lines and the secondary ones as pink dots. They are associated with the BCPs. Carbon and hydrogen atoms are shown in black and gray, respectively. The contours (*e*a_o_^−3^) are at 2^*l*^ (*l* = ±8, ±7,…, and 0).

The structures of **1** (*C*_*i*_) and **2** (*C*_1_) determined by the X-ray analysis are denoted by **1** (*C*_*i*_)_obsd_ and **2** (*C*_1_)_obsd_, respectively [39]. The structural parameters are shown in Tables S2 and S3 in Supplementary File, respectively.  Figure 3 contains the selected structural parameters for **1** (*C*_*i*_)_obsd_. The structures are optimized for G(**A**) of H_2_Br_4_ (*C*_2h_), Me_2_Br_4_ (*C*_2h_), Br_6_ (*C*_2h_), and Br_6_ (*C*_2_) and G(**B**) of H_4_Se_2_Br_6_ (*C*_*i*_), Me_4_Se_2_Br_6_ (*C*_*i*_), **5** (*C*_*i*_), and **6** (*C*_*i*_), together with **3** (*C*_s_), **4** (*C*_s_), **7** (*C*_2h_), **8** (*C*_2h_), and Br_2_ (*D*_∞h_). The optimized structural parameters are also collected in Tables S2 and S3 in Supplementary File. The frequency analysis was successful for the optimized structures, except for **1** (*C*_*i*_)_obsd_ and Br_6_ (*C*_2h_). All positive frequencies were obtained for **1** (*C*_*i*_), if calculated with CAM-B3LYP/BSS-A, which confirms the structure. The Br---Br distances of Br_4_*σ*(4c–6e) in **1** (*C*_*i*_) are somewhat longer if optimized at the CAM-B3LYP level, relative to **1** (*C*_*i*_)_obsd_. While one imaginary frequency is detected in Br_6_ (*C*_2h_), Br_6_ (*C*_2_) has all positive frequencies. The optimized structures are not shown in figures, instead, some of them can be found in Figures 3 and 5, where the molecular graphs are drawn on the optimized structures.  Figure 3 contains the optimized *r*(^A^Br-^A^Br) and *r*(^A^Br-^B^Br) distances for the models and the charge developed at ^B^Br in the original R-^B^Br and Br-(R_2_)Se-^B^Br (*Qn* (^B^Br)), which give the models of G(**A**) and G(**B**), respectively. The *r*(^A^Br-^B^Br) values become shorter in the order shown in equation (7), if evaluated with MP2/BSS-A:

(7) rBAr−BBr:H2Br4C2h>1CiCAM>Br6C2 and C2h>Br6C2hobsd40>Me2Br4C2h>Br42−D∞h>1Ciobsd≥Me4Se2Br6Ci≥H4Se2Br6Ci>5Ci≥6Ci.

One imaginary frequency was also predicted for Br_4_^2−^ (*D*_∞h_) if optimized with MP2/BSS-A. Br_4_^2−^ (*D*_∞h_) seems to collapse to Br_3_^−^ and Br^−^, according to the imaginary frequency. The double negative charges in Br_4_^2−^ (*D*_∞h_) would be responsible for the results. The electrostatic repulsion between the double negative charges will operate to collapse it.

### 3.2. Energies for Formation of Br_4_*σ*(4c–6e) and NBO Analysis

Energies for the formation of R′**Br**_**4**_R′ from the components (2R′**Br** + **Br**_**2**_) (Δ*E*) are defined by equation (8). The Δ*E* values evaluated on the energy surface are denoted by Δ*E*_ES_, while those corrected with the zero-point energies are by Δ*E*_ZP_. The Δ*E*_ES_ and Δ*E*_ZP_ values for the optimized structures are given in Table S2 in Supplementary File. Δ*E*_ZP_ are excellently correlated to Δ*E*_ES_ (Δ*E*_ZP_ = 0.99Δ*E*_ES_ + 1.93: *R*_c_^2^ = 0.9998, see Figure S3 in Supplementary File):

(8) $$\begin{array}{c} \Delta E\left(R_{2}^{\prime }{\text{Br}}_{4}\right)=E\left(R_{2}^{\prime }{\text{Br}}_{4}\right)-\left[2E\left(R^{\prime }\text{Br}\right)+E\left({\text{Br}}_{2}\right)\right], \end{array}$$

(9) $$\begin{array}{c} E\left(2\right)=q_{i}\times \frac{F{\left(i,j\right)}^{2}}{\left(\varepsilon_{j}-\varepsilon_{i}\right)}. \end{array}$$

NBO analysis [62] was applied to ^A^Br---^B^Br of the species to evaluate the contributions from CT to stabilize R′-^B^Br---^A^Br-^A^Br---^B^Br-R′. For each donor NBO (*i*) and acceptor NBO (*j*), the stabilization energy *E*(2) is calculated based on the second-order perturbation theory in NBO, according to equation (9), where *q*_*i*_ is the donor orbital occupancy, *ε*_*i*_ and *ε*_*j*_ are diagonal elements (orbital energies), and *F*(*i*, *j*) is the off-diagonal NBO Fock matrix element. The results are collected in Table S4 in Supplementary File. The Δ*E*_ES_ values are very well correlated to *E*(2) for the optimized structures, except for Br_4_^2−^ (*D*_∞h_). (Δ*E*_ES_ = –0.71(2*E*(2)) + 7.17: *R*_*c*_^2^ = 0.959, see Figure S4 in Supplementary File). Br_4_^2−^ (*D*_∞h_) is predicted to be less stable than the components.

Before application of QTAIM-DFA to Br_4_*σ*(4c–6e) and Se_2_Br_5_*σ*(7c–10e), molecular graphs were examined, as shown in the next section.

### 3.3. Molecular Graphs with Contour Plots for the Species Containing Br_4_*σ*(4c–6e), Se_2_Br_5_*σ*(7c–10e), and Related Linear Interactions

Figure 5 illustrates the molecular graphs of **5** (*C*_*i*_), **6** (*C*_*i*_), **7** (*C*_2h_), and **8** (*C*_2h_), drawn on the optimized structures, together with **1** (*C*_*i*_)_obsd_ and **2** (*C*_1_)_obsd_. Figure 5 also shows the contour plots of *ρ*(*r*) drawn on the suitable plane in the molecular graphs. BCPs are well demonstrated to locate on the (three-dimensional) saddle points of *ρ*(*r*). Molecular graphs of Me_2_Br_4_ (*C*_2h_), Br_6_ (*C*_2_), Br_4_^2−^ (*D*_∞h_), and Br(Me_2_)SeBr_4_Se(Me_2_)Br (*C*_*i*_) are shown in Figure 3, which are drawn on the optimized structures.

### 3.4. Survey of Br_4_*σ*(4c–6e) and Se_2_Br_5_*σ*(7c–10e)

BPs in Br_4_*σ*(4c–6e) and Se_2_Br_6_*σ*(7c–10e) seem straight, as shown in Figures 3 and 5. To show the linearity more clearly, the lengths of BPs (*r*_BP_) for Br_4_*σ*(4c–6e) are calculated. The values are collected in Table S5 in Supplementary File, together with the corresponding straight-line distances (*R*_SL_). The table contains the values for Se_2_Br_6_*σ*(7c–10e) in **7** (*C*_2h_) and **8** (*C*_2h_). The differences between them (Δ*r*_BP_ = *r*_BP_–*R*_SL_) are less than 0.003 Å. The *r*_BP_ values are plotted versus *R*_SL_, which are shown in Figure S5 in Supplementary File. The correlations are excellent, as shown in the figure. Therefore, Br_4_*σ*(4c–6e) and Se_2_Br_6_*σ*(7c–10e) in the species can be approximated by the straight lines.

QTAIM functions are calculated for Br_4_*σ*(4c–6e) at BCPs.  Table 1 collects the values for the interactions. *H*_b_(**r**_c_) is plotted versus *H*_b_(**r**_c_) − *V*_b_(**r**_c_)/2 for the data shown in Table 1, together with those from the perturbed structures generated with CIV. Figure 4 shows the plots for the ^A^Br-∗-^A^Br and ^A^Br-∗-^B^Br interactions in Br_4_*σ*(4c–6e) of the bromine species. The plots for ^A^Br-∗-^A^Br appear in the region of *H*_b_(**r**_c_) − *V*_b_(**r**_c_)/2 > 0 and *H*_b_(**r**_c_) < 0, for all species, except for the original Br_2_ (*D*_∞h_), of which the plot appears in the region of *H*_b_(**r**_c_) − *V*_b_(**r**_c_)/2 < 0 and *H*_b_(**r**_c_) < 0. Therefore, the interactions are all classified by the *regular*-CS (closed shell) interactions, except for Br_2_ (*D*_∞h_), which is classified by the SS (shard shell) interaction. On the contrary, data of ^A^Br-∗-^B^Br appear in the region of *H*_b_(**r**_c_) − *V*_b_(**r**_c_)/2 > 0 and *H*_b_(**r**_c_) > 0 for all species, except for those in H_4_Se_2_Br_6_ (*C*_*i*_), Me_4_Se_2_Br_6_ (*C*_*i*_), **5** (*C*_*i*_), and **6** (*C*_*i*_), which appear in the region of *H*_b_(**r**_c_) − *V*_b_(**r**_c_)/2 > 0 and *H*_b_(**r**_c_) < 0. As a result, ^A^Br-∗-^B^Br is classified by the *pure*-CS interactions (*p*-CS) for all, except for the four species, of which ^A^Br-∗-^B^Br is classified by the *regular*-CS interactions (*r*-CS). The ^A^Br-∗-^B^Br interaction in Br_4_^2−^ (*D*_∞h_) is very close to the borderline between *p*-CS and *r*-CS since *H*_b_(**r**_c_) = 0.0001 au for Br_4_^2−^ (*D*_∞h_), which is very close to zero. QTAIM-DFA parameters of (*R*, *θ*) and (*θ*_p_, *κ*_p_) are obtained by analysing the plots of *H*_b_(**r**_c_) versus *H*_b_(**r**_c_) − *V*_b_(**r**_c_)/2 in Figure 4, according to equations (S3)–(S6).  Table 1 collects the QTAIM-DFA parameters for Br_4_*σ*(4c–6e). The classification of interactions will also be discussed based on the (*R*, *θ*) values.

QTAIM functions are similarly calculated for Se_2_Br_6_*σ*(7c–10e) at BCPs, together with the related interactions. *H*_b_(**r**_c_) is similarly plotted versus *H*_b_(**r**_c_) − *V*_b_(**r**_c_)/2 although not shown in the figures. Then, QTAIM-DFA parameters of (*R*, *θ*) and (*θ*_p_, *κ*_p_) are obtained by analysing the plots, according to equations (S3)–(S6).  Table 2 collects the QTAIM-DFA parameters of (*R*, *θ*) and (*θ*_p_, *κ*_p_) for Br_4_*σ*(4c–6e).

### 3.5. Nature of Br_4_*σ*(4c–6e)

Interactions are characterized by (*R*, *θ*), which correspond to the data from the fully optimized structures. On the contrary, they are characterized employing (*θ*_p_, *κ*_p_) derived from the data of the perturbed structures around the fully optimized structures and the fully optimized ones. In this case, the nature of interactions is substantially determined based of the (*R*, *θ*, *θ*_p_) values, while the *κ*_p_ values are used only additionally. It is instructive to survey the criteria before detail discussion. The criteria tell us that 180° < *θ* (*H*_b_(**r**_c_) − *V*_b_(**r**_c_)/2 < 0) for the SS interactions, 90° < *θ* < 180° (*H*_b_(**r**_c_) < 0) for the *r*-CS interactions, and 45° < *θ* < 90° (*H*_b_(**r**_c_) > 0) for *p*-CS interactions. The *θ*_p_ value characterizes the interactions. In the *p*-CS region of 45° < *θ* < 90°, the character of interactions will be the vdW type for 45° < *θ*_p_ < 90°, whereas it will be the typical HB type without covalency (*t*-HB_nc_) for 90° < *θ*_p_ < 125°, where *θ*_p_ = 125° is tentatively given for *θ* = 90°. The CT interaction will appear in the *r*-CS region of 90° < *θ* < 180°. The *t*-HB type with covalency (*t*-HB_wc_) appears in the region of 125° < *θ*_p_ < 150° (90° < *θ* < 115°), where (*θ*, *θ*_p_) = (115°, 150°) is tentatively given as the borderline between *t*-HB_wc_ and the CT-MC nature. The borderline for the interactions between CT-MC and CT-TBP types is defined by *θ*_p_ = 180°. *θ* = 150° is tentatively given for *θ*_p_ = 180°. Classical chemical bonds of SS (180° < *θ*) will be strong (Cov-s) when *R* > 0.15 au, whereas they will be weak (Cov-w) for *R* < 0.15 au. The classification and characterization of interactions are summarized in Table S1 and Scheme S3 in Supplementary File.

The ^A^Br-∗-^A^Br and ^A^Br-∗-^B^Br interactions of Br_4_*σ*(4c–6e) will be classified and characterized based on the (*R*, *θ*, *θ*_p_) values, employing the standard values as a reference (see Scheme S2 in Supplementary File). *R* < 0.15 au for all interactions in Table 1; therefore, no Cov-s were detected in this work. The (*θ*, *θ*_p_) values are (180.1°, 191.8°) for the original Br_2_ (*D*_∞h_) if evaluated with MP2/BSS-A. Therefore, the nature of Br-∗-Br in Br_2_ (*D*_∞h_) is classified by the SS interactions and characterized as the Cov-w nature, which is denoted by SS/Cov-w. The (*θ*, *θ*_p_) values are (170.6–179.0°, 190.6–191.7°) for ^A^Br-∗-^A^Br of Br_4_*σ*(4c–6e) in the optimized structures in Table 1, of which nature is *r*-CS/CT-TBP. The (*θ*, *θ*_p_) values are (78.0–84.1°, 94.7–105.1°) for ^A^Br-∗-^B^Br in the optimized structures of Br_6_ (*C*_2_), Br_6_ (*C*_2h_), and R_2_Br_4_ (*C*_2h_) (R = H and Me); therefore, the nature is predicted to be *r*-CS/*t*-HB_wc_. The nature of ^A^Br-∗-^B^Br in R_4_Se_2_Br_6_ (*C*_*i*_) (R = H and Me), **5** (*C*_*i*_) and **6** (*C*_*i*_), is *r*-CS/*t*-HB_wc_, judging from the (*θ*, *θ*_p_) values of (90.9–92.8°, 116.4–122.5°). The calculated (*θ*, *θ*_p_) values of ^A^Br-∗-^A^Br and ^A^Br-∗-^B^Br for the optimized structure of Br_4_^2−^ (*D*_∞h_) are (170.6°, 190.6°) and (89.5°, 118.2°), respectively. In this case, ^A^Br-∗-^A^Br and ^A^Br-∗-^B^Br are predicted to have the nature of *r*-CS/CT-TBP and *p*-CS/*t*-HB_nc_, respectively. However, ^A^Br-∗-^B^Br is just the borderline region to the *r*-CS interactions with *θ* = 89.5°. The characteristic nature of the ^B^E---^A^E-^A^E---^B^E interactions in Br_4_^2−^ (*D*_∞h_) would be controlled by the double negative charges in the species.

The results in Table 1 show that the ^A^Br-∗-^A^Br interaction in Br_4_*σ*(4c–6e) becomes weaker, as the strength of the corresponding ^A^Br-∗-^B^Br increases. The strength of ^A^Br-∗-^A^Br becomes weaker in the order shown in equation (10), if evaluated by *θ*, while that of ^A^Br-∗-^B^Br increases in the order shown in equation (11), if measured by *θ*. Very similar results were obtained by *θ*_p_:

(10) $$\begin{array}{c} \theta \text{ for}{\;}^{\text{A}}\text{Br}-*{-}^{A}\text{Br}:\\ {\text{Br}}_{2}\left(D_{\infty h}\right)>{\text{H}}_{2}{\text{Br}}_{4}\left(C_{2h}\right)\geq {\text{Br}}_{6}\left(C_{2}\text{ and }C_{2h}\right)\\ >{\text{Me}}_{2}{\text{Br}}_{4}\left(C_{2h}\right)>{\text{H}}_{4}{\text{Se}}_{2}{\text{Br}}_{6}\left(C_{i}\right)\geq {\text{Me}}_{4}{\text{Se}}_{2}{\text{Br}}_{6}\left(C_{i}\right)\\ \geq 1{\left(C_{i}\right)}_{\text{obsd}}>5\left(C_{i}\right)>6\left(C_{i}\right)>{\text{Br}}_{6}{\left(C_{2h}\right)}_{\text{obsd}}, \end{array}$$

(11) $$\begin{array}{c} \theta \text{ for}{\;}^{\text{A}}\text{Br}-*{-}^{\text{B}}\text{Br}:\\ {\text{H}}_{2}{\text{Br}}_{4}\left(C_{2h}\right)>{\text{Br}}_{6}\left(C_{2h}\text{ and }C_{2}\right)\geq {\text{Br}}_{6}{\left(C_{2h}\right)}_{\text{obsd}}\\ >{\text{Me}}_{2}{\text{Br}}_{4}\left(C_{2h}\right)<1{\left(C_{i}\right)}_{\text{obsd}}<{\text{Me}}_{4}{\text{Se}}_{2}{\text{Br}}_{6}\left(C_{i}\right)\\ <{\text{H}}_{4}{\text{Se}}_{2}{\text{Br}}_{6}\left(C_{i}\right)<5\left(C_{i}\right)\approx 6\left(C_{i}\right). \end{array}$$

The orders shown in equations (10) and (11) seem to reasonably explain the characteristic behavior of Br_4_*σ*(4c–6e). The results must be the reflection of the *n*_p_(^B^Br) ⟶ *σ*∗(^A^Br-^A^Br) ← *n*_p_(^B^Br) form of Br_4_*σ*(4c–6e), where ^A^Br-∗-^A^Br and ^A^Br-∗-^B^Br become weaker and stronger, respectively, as the CT interaction increases. Br_4_*σ*(4c–6e) will be stabilized more effectively, if the negative charge is developed more at ^B^Br. However, the two Br^−^ ligands in Br_4_^2−^ (*D*_∞h_) seem not so effective than that expected. This would come from the electrostatic repulsive factor between the double negative charges in Br_4_^2−^ (*D*_∞h_), as mentioned above.

The *θ* values for (^A^Br-∗-^A^Br and ^A^Br-∗-^B^Br) in Br_6_ (*C*_2h_)_obsd_ and **1** (*C*_*i*_)_obsd_ are (165.2°, 82.5°) and (175.3°, 87.7°), respectively. Therefore, ^A^Br-∗-^A^Br and ^A^Br-∗-^B^Br are classified by *r*-CS and *p*-CS, respectively. Both ^A^Br-∗-^A^Br and ^A^Br-∗-^B^Br in Br_6_ (*C*_2h_)_obsd_ are predicted to be weaker than those in **1** (*C*_*i*_)_obsd_, respectively. The results would be curious at the first glance, since ^A^Br-∗-^A^Br will be weaker, if ^A^Br-∗-^B^Br in ^B^Br-∗-^A^Br-∗-^A^Br-∗-^B^Br becomes stronger, as mentioned above. They would be affected from the surrounding, such as the crystal packing effect. A Br_2_ molecule interacts with four bromine atoms adjacent to the Br_2_ molecule on the *bc*-plane in crystals, equivalently with 3.251 Å [40].

Similar investigations were carried out for I_4_*σ*(4c–6e), which will be discussed elsewhere (it is demonstrated that Br_4_*σ*(4c–6e) is predicted to be somewhat stronger than I_4_*σ*(4c–6e)).

### 3.6. Nature of Se_2_Br_5_*σ*(7c–10e)

**Figure 6:**
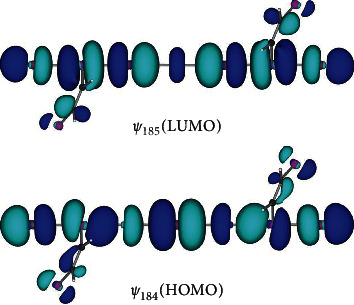
Molecular orbitals for *σ*(7c–10e). *ψ*_184_ (HOMO) and *ψ*_185_ (LUMO) of **8** (*C*_2h_).

The nature of Se_2_Br_5_*σ*(7c–10e) in **7** (*C*_2h_) and **8** (*C*_2h_) is elucidated, together with SeBr_2_*σ*(3c–4e) in **3** and SeBr_4_*σ*(4c–6e) in **4**. The results are collected in Table 2.  Figure 6 shows symmetric *ψ*_184_ (HOMO) and antisymmetric *ψ*_185_ (LUMO) of **8** (*C*_2h_), which correspond to *ψ*_5_ and *ψ*_6_ in *σ*(7c–10e), illustrated in Figure 1 although the Se atoms are contained in the linear Se_2_Br_5_*σ*(7c–10e) in **8** (*C*_2h_). The linear seven atomic orbitals on Se_2_Br_5_ are shown to construct *ψ*_184_ (HOMO) and *ψ*_185_ (LUMO) of **8** (*C*_2h_), which can be analysed as the Se_2_Br_5_*σ*(7c–10e) [39], so can the linear interaction in **7** (*C*_2h_), although not shown. The pseudolinear interaction of the seven atoms of **1** (*C*_1_)_obsd_ could also be explained by the Se_2_Br_5_*σ*(7c–10e) model.

The results demonstrate that Se_2_Br_5_*σ*(7c–10e) stabilize well **7** (*C*_2h_) and **8** (*C*_2h_) although **1** (*C*_1_)_obsd_ seems not so effective. The negative charge developed at the Br atom in **3** would not be sufficient to stabilize Se_2_Br_5_*σ*(7c–10e) in **1** (*C*_1_)_obsd_, relative to the case of the Br^−^ anion in **7** (*C*_2h_) and **8** (*C*_2h_), irrespective of the highly negatively charged Br atoms in SeBr_2_*σ*(3c–4e) of **3**.

## 4. Conclusion

The intrinsic dynamic and static nature of Br_4_*σ*(4c–6e) is elucidated for **1** (*C*_*i*_)_obsd_ and the related species with QTAIM-DFA, employing the perturbed structures generated with CIV. The ^A^Br-^A^Br interactions in ^B^Br-∗-^A^Br-∗-^A^Br-∗-^B^Br of Br_4_*σ*(4c–6e) are weaker than Br-∗-Br in the optimized structure of Br_2_ (*D*_∞h_), which is predicted to have the SS/Cov-w nature. The ^A^Br-^A^Br interactions in Br_4_*σ*(4c–6e) of the models are predicted to have the *r*-CS/CT-TBP nature, if optimized with MP2/BSS-A. The ^A^Br-^A^Br interaction in **1** (*C*_*i*_)_obsd_ also appears in the *r*-CS region. On the contrary, the ^A^Br-^B^Br interactions in Br_6_ (*C*_2_), Br_6_ (*C*_2h_), H_2_Br_4_ (*C*_2h_), and Me_2_Br_4_ (*C*_2h_) are predicted to have the *p*-CS/*t*-HB_nc_ nature, whereas those in H_4_Se_2_Br_4_ (*C*_*i*_), Me_4_Se_2_Br_4_ (*C*_*i*_), **5** (*C*_*i*_), and **6** (*C*_*i*_) have the *r*-CS/*t*-HB_wc_ nature, if evaluated with MP2/BSS-A. The ^A^Br-∗-^B^Br interactions become stronger in the order of H_2_Br_4_ (*C*_2h_) < Br_6_ (*C*_2h_) ≤ Br_6_ (*C*_2_) < Me_2_Br_4_ (*C*_2h_) << Me_4_Se_2_Br_6_ (*C*_*i*_) ≤ H_4_Se_2_Br_6_ (*C*_*i*_) ≤ **5** (*C*_*i*_) < **6** (*C*_*i*_), which is the inverse order for ^A^Br-∗-^A^Br, as a whole. The results are in accordance with the CT interaction of the *n*_p_(^B^Br) ⟶ *σ*∗(^A^Br-^A^Br) ← *n*_p_(^B^Br) form derived from Br_4_*σ*(4c–6e). The decreased binding force of ^A^Br-∗-^A^Br must be transferred to ^A^Br-∗-^B^Br in Br_4_*σ*(4c–6e). Namely, it is demonstrated that Br_4_*σ*(4c–6e) is stabilized as the strength of ^A^Br-∗-^B^Br in Br_4_*σ*(4c–6e) increases, while ^A^Br-∗-^A^Br becomes weakened relative to that in the original Br_2_ (*D*_∞h_). In this process, Br_4_*σ*(4c–6e) is totally stabilized. The ^A^Br-∗-^A^Br and ^A^Br-∗-^B^Br interactions in Br_6_ (*C*_2h_)_obsd_ and **1** (*C*_*i*_)_obsd_ are classified by the *r*-CS and *p*-CS interactions, respectively, where the interactions in Br_6_ (*C*_2h_)_obsd_ seem somewhat weaker than those in **1** (*C*_*i*_)_obsd_. The Se_2_Br_5_*σ*(7c–10e) interactions are similarly elucidated for **2** (*C*_1_)_obsd_ and the anionic models of **7** (*C*_2h_) and **8** (*C*_2h_). The Se_2_Br_5_*σ*(7c–10e) nature is clearly established for the optimized structures of **7** (*C*_2h_) and **8** (*C*_2h_), rather than **2** (*C*_1_)_obsd_. Extended hypervalent interactions of the *σ*(*m*c–*n*e: 4 ≤ *m*; *m* < *n* < 2*m*) type are shown to be well analysed and evaluated with QTAIM-DFA, employing the perturbed structures generated with CIV, exemplified by Br_4_*σ*(4c–6e) and Se_2_Br_5_*σ*(7c–10e).

## Figures and Tables

**Table 1:** QTAIM functions and QTAIM-DFA parameters for ^B^Br-∗-^A^Br-∗-^A′^Br-∗-^B′^Br at BCPs in Br_4_*σ*(4c–6e), together with ^A^Br-∗-^A^Br in Br_2_, evaluated with MP2/BSS-A^a)^.

Species (symmetry)	Interaction X-∗-Y	*ρ*_b_(**r**_c_) (*e*a_o_^−3^)	*c*∇^2^*ρ*_b_(**r**_c_)^b)^ (au)	*H*_b_(**r**_c_) (au)	*k*_b_(**r**_c_)^c)^	*R* ^d)^ (au)	*θ* ^e)^ (°)	*C*_*ij*_ (Å mdyn^−1^)	*θ*_p:CIV_^f)^ (°)	*κ*_p:CIV_^g)^ (au^−1^)	Predicted nature
Br_2_ (*D*_∞h_)^h)^	Br-∗-Br	0.1130	−0.0001	−0.0497	−2.005	0.0497	180.1	0.4	191.8	1.8	SS/Cov-w^i)^
Br_4_^2−^ (*D*_∞h_)^j)^	^A^Br-∗-^A^Br	0.0922	0.0052	−0.0313	−1.751	0.0317	170.6	0.8	190.6	3.6	*r*-CS/CT-TBP^k)^
	^**A**^ **Br-∗-** ^**B**^ **Br**	0.0198	0.0058	0.0001	−0.995	0.0058	89.5	−19.6	118.2	146	*p*-CS/*t*-HB_nc_^l)^
Br_6_ (*C*_2_)	^A^Br-∗-^A^Br	0.1099	0.0010	−0.0466	−1.960	0.0467	178.8	0.4	191.3	2.3	*r*-CS/CT-TBP^k)^
	^A^Br-∗-^B^Br	0.0131	0.0049	0.0009	−0.899	0.0050	79.6	14.3	97.8	105	*p*-CS/*t*-HB_nc_^l)^
Br_6_ (*C*_2h_)^m)^	^A^Br-∗-^A^Br	0.1099	0.0010	−0.0466	−1.961	0.0467	178.8	0.4	191.7	1.7	*r*-CS/CT-TBP^k)^
	^A^Br-∗-^B^Br	0.0131	0.0049	0.0009	−0.899	0.0050	79.6	14.3	97.6	97	*p*-CS/*t*-HB_nc_^l)^
Br_6_ (*C*_2h_)_obsd_^n)^	^A^Br-∗-^A^Br	0.0765	0.0053	−0.0200	−1.654	0.0207	165.2				*r*-CS
	^A^Br-∗-^B^Br	0.0156	0.0055	0.0007	−0.929	0.0055	82.5				*p*-CS
H_2_Br_4_ (*C*_2h_)	^A^Br-∗-^A^Br	0.1101	0.0008	−0.0468	−1.966	0.0468	179.0	0.4	191.7	2.0	*r*-CS/CT-TBP^k)^
	^A^Br-∗-^B^Br	0.0118	0.0045	0.0010	−0.881	0.0046	78.0	15.5	94.7	100	*p*-CS/*t*-HB_nc_^l)^
Me_2_Br_4_	^A^Br-∗-^A^Br	0.1076	0.0016	−0.0444	−1.932	0.0445	177.9	0.4	191.4	1.8	*r*-CS/CT-TBP^k)^
	^A^Br-∗-^B^Br	0.0164	0.0057	0.0006	−0.945	0.0057	84.1	10.5	105.1	100	*p*-CS/*t*-HB_nc_^l)^
H_4_Se_2_Br_6_ (*C*_*i*_)	^A^Br-∗-^A^Br	0.1028	0.0031	-0.0400	−1.866	0.0401	175.6	0.5	191.5	2.5	*r*-CS/CT-TBP^k)^
	^A^Br-∗-^B^Br	0.0220	0.0068	−0.0002	−1.016	0.0068	91.9	9.9	117.4	75	*r*-CS/*t*-HB_wc_^o)^
Me_4_Se_2_Br_6_ (*C*_*i*_)	^A^Br-∗-^A^Br	0.1028	0.0032	−0.0400	−1.862	0.0402	175.4	0.5	191.4	3.7	*r*-CS/CT-TBP^k)^
	^A^Br-∗-^B^Br	0.0212	0.0067	−0.0001	−1.008	0.0067	90.9	9.9	116.4	101	*r*-CS/*t*-HB_wc_^o)^
**5** (*C*_*i*_)^p)^	^A^Br-∗-^A^Br	0.1016	0.0036	−0.0389	−1.844	0.0391	174.7	0.5	191.5	4.4	*r*-CS/CT-TBP^k)^
	^A^Br-∗-^B^Br	0.0226	0.0070	−0.0003	−1.023	0.0070	92.7	6.8	118.0	557	*r*-CS/*t*-HB_wc_^o)^
	^A^Br-∗-^B^Br^q)^	0.0226	0.0070	−0.0003	−1.023	0.0070	92.7	6.8	118.0	551	*r*-CS/*t*-HB_wc_^o)^
**5** (*C*_*i*_)^r)^	^A^Br-∗-^A^Br	0.1047	0.0020	−0.0383	−1.905	0.0384	177.0	0.5	191.3	2.5	*r*-CS/CT-TBP^k)^
	^A^Br-∗-^B^Br	0.0145	0.0048	0.0008	−0.904	0.0048	80.1	15.9	97.4	102	*p*-CS/*t*-HB_nc_^o)^
**6** (*C*_*i*_)	^A^Br-∗-^A^Br	0.1014	0.0037	−0.0388	−1.841	0.0389	174.6	0.5	191.5	36	*r*-CS/CT-TBP^k)^
	^A^Br-∗-^A^Br^q)^	0.1014	0.0037	−0.0388	−1.841	0.0389	174.6	0.5	191.6	36	*r*-CS/CT-TBP^k)^
	^A^Br-∗-^B^Br^q)^	0.0227	0.0070	−0.0004	−1.024	0.0071	92.8	42.1	122.5	2474	*r*-CS/*t*-HB_wc_^o)^
**6** (*C*_*i*_)^r)^	^A^Br-∗-^A^Br	0.1044	0.0021	−0.0380	−1.901	0.0381	176.9	0.5	191.3	2.6	*r*-CS/CT-TBP^k)^
	^A^Br-∗-^B^Br	0.0147	0.0048	0.0008	−0.907	0.0049	80.3	16.6	97.8	103	*p*-CS/*t*-HB_nc_^o)^
**1** (*C*_*i*_)^r)^	^A^Br-∗-^A^Br	0.1063	0.0013	−0.0398	−1.939	0.0398	178.1	0.5	191.7	2.3	*r*-CS/CT-TBP^k)^
	^A^Br-∗-^B^Br	0.0123	0.0043	0.0010	−0.868	0.0044	76.9	18.3	91.8	100	*p*-CS/*t*-HB_nc_^l)^
**1** (*C*_*i*_)_obsd_^s)^	^A^Br-∗-^A^Br	0.1019	0.0032	−0.0393	−1.860	0.0394	175.3				*r*-CS
	^A^Br-∗-^B^Br	0.0200	0.0066	0.0003	−0.979	0.0066	87.7				*p*-CS

^a)^See the text for BSS. ^b)^*c*∇^2^*ρ*_b_(**r**_c_) = *H*_b_(**r**_c_) − *V*_b_(**r**_c_)/2, where *c* = *ћ*^2^/8*m*. ^c)^*k*_b_(**r**_c_) = *V*_b_(**r**_c_)/*G*_b_(**r**_c_). ^d)^*R* = (*x*^2^ + *y*^2^)^1/2^, where (*x*, *y*) = (*H*_b_(**r**_c_) − *V*_b_(**r**_c_)/2, *H*_b_(**r**_c_)). ^e)^*θ* = 90° − tan^−1^ (*y*/*x).*^f)^*θ*_p_ = 90°– tan^−1^(d*y*/d*x*). ^g)^*κ*_p_ = |d^2^*y*/d*x*^2^|/[1 + (d*y*/d*x*)^2^]^3/2^. ^h)^The Br-Br distance in Br_2_ was optimized to be 2.2756 Å with MP2/BSS-A, which was very close to the observed distance in the gas phase (2.287 Å) [63]. However, the values are shorter than those determined by the X-ray crystallographic analysis (2.491 Å) [40] by 0.210 Å. The noncovalent Br---Br distance is 3.251 Å in crystal, which is shorter than the sum of the van der Waals radii [64] by 0.45 Å. ^i)^The SS interaction of the weak covalent nature. ^j)^With one imaginary frequency for the vibration mode of the SGU symmetry. ^k)^The *regular*-CS interaction of the CT-TBP nature. ^l)^The *pure*-CS interaction of the HB nature with no covalency. ^m)^With one imaginary frequency for the rotational mode around the linear Br_4_ interaction. ^n)^See ref. [40] ^o)^The *regular*-CS interaction of the HB nature with covalency. ^p)^With one imaginary frequency for the vibration mode of the AU symmetry. ^q)^*w* = (0), ±0.025, and ±0.05. ^r)^At the CAM-B3LYP level. ^s)^See ref. [39].

**Table 2:** QTAIM functions and QTAIM-DFA parameters for ^A^Br-∗-^A^Se-∗-^B^Br-∗-^C^Br-∗-^D^Br-∗-^B^Se-∗-^E^Br at BCPs in **7** (*C*_2h_), **8** (*C*_2h_), and **2** (*C*_1_)_obsd_, together with ^A^Br-∗-^A^Se-∗-^B^Br in **3** (*C*_s_) and ^A^Br-∗-^A^Se-∗-^B^Br-∗-^C^Br-∗-^D^Br in **4** (*C*_s_), evaluated with MP2 BSS‐A^a)^.

Species (symmetry)	Interaction X-∗-Y	*ρ*_b_(**r**_c_) (*e*a_o_^−3^)	*c*∇^2^*ρ*_b_(**r**_c_)^b)^ (au)	*H*_b_(**r**_c_) (au)	*k*_b_(**r**_c_)^c)^	*R* ^d)^ (au)	*θ* ^e)^ (°)	*C*_*ij*_ (Å mdyn^−1^)	*θ*_p:CIV_^f)^ (°)	*κ*_p:CIV_^g)^ (au^−1^)	Predicted nature
**7** (*C*_2h_)	^A^Se-∗-^A^Br^h)^	0.0423	0.0080	−0.0056	−1.258	0.0098	124.8	6.3	169.9	55	*r*-CS/CT-MC^i)^
	^A^Se-∗-^B^Br^j)^	0.0825	0.0043	−0.0264	−1.753	0.0267	170.7	1.2	192.2	2.2	*r*-CS/CT-TBP^k)^
	^B^Br-∗-^C^Br^l)^	0.0335	0.0086	−0.0022	−1.115	0.0088	104.6	9.4	145.5	102	*r*-CS/*t*-HB_wc_^m)^
**8** (*C*_2h_)	^A^Se-∗-^A^Br^h)^	0.0492	0.0085	−0.0079	−1.318	0.0116	133.0	2.3	172.7	53	*r*-CS/CT-MC^i)^
	^A^Se-∗-^B^Br^j)^	0.0662	0.0075	−0.0158	−1.513	0.0175	154.6	2.2	187.7	17	*r*-CS/CT-TBP^k)^
	^B^Br-∗-^C^Br^l)^	0.0398	0.0092	−0.0038	−1.171	0.0100	112.5	4.2	151.5	54	*r*-CS/*t*-HB_wc_^m)^
**2** (*C*_1_)_obsd_^n)^	^A^Br-∗-^A^Se	0.0219	0.0065	−0.0005	−1.039	0.0065	94.6				*r*-CS
	^A^Se-∗-^B^Br	0.0576	0.0102	−0.0113	−1.356	0.0152	137.8				*r*-CS
	^B^Br-∗-^C^Br	0.0952	0.0068	−0.0337	−1.713	0.0343	168.6				*r*-CS
	^C^Br-∗-^D^Br	0.0183	0.0062	0.0005	−0.961	0.0062	85.7				*p*-CS
	^D^Br-∗-^B^Se	0.0818	0.0063	−0.0271	−1.682	0.0278	166.9				*r*-CS
	^B^Se-∗-^E^Br	0.0753	0.0072	−0.0220	−1.604	0.0231	161.9				*r*-CS
**3** (*C*_s_)	^A^Br-∗-^A^Se	0.0737	0.0061	−0.0214	−1.636	0.0223	164.0	0.8	185.8	8.4	*r*-CS/CT-TBP^k)^
	^A^Se-∗-^B^Br	0.0678	0.0069	−0.0177	−1.562	0.0189	158.7	1.0	183.0	18	*r*-CS/CT-TBP^k)^
**4** (*C*_s_)	^A^Br-∗-^A^Se	0.0131	0.0042	0.0004	−0.945	0.0042	84.0	6.4	105.1	84	*p*-CS/*t*-HB_nc_^o)^
	^A^Se-∗-^B^Br	0.0425	0.0088	−0.0052	−1.229	0.0103	120.6	4.6	163.2	63	*r*-CS/CT-MC^i)^
	^B^Br-∗-^C^Br	0.0933	0.0059	−0.0321	−1.732	0.0326	169.6	0.9	192.1	5.6	*r*-CS/CT-TBP^k)^

^a)^See the text for BSS. ^b)^*c*∇^2^*ρ*_b_(**r**_c_) = *H*_b_(**r**_c_) − *V*_b_(**r**_c_)/2, where *c* = *ћ*^2^/8*m*. ^c)^*k*_b_(**r**_c_) = *V*_b_(**r**_c_)/*G*_b_(**r**_c_). ^d)^*R* = (*x*^2^ + *y*^2^)^1/2^, where (*x*, *y*) = (*H*_b_(**r**_c_) − *V*_b_(**r**_c_)/2, *H*_b_(**r**_c_)). ^e)^*θ* = 90° − tan^−1^ (*y*/*x*). ^f)^*θ*_p_ = 90° − tan^−1^ (d*y*/d*x*). ^g)^*κ*_p_ = |d^2^*y*/d*x*^2^|/[1 + (d*y*/d*x*)^2^]^3/2^. ^h)^Because it has *C*_*i*_ symmetry, it is the same as ^B^Se-∗-^E^Br. ^i)^The *regular*-CS interaction of the CT-MC nature. ^j)^The same as ^B^Se-∗-^D^Br. ^k)^The *regular*-CS interaction of the CT-TBP nature. ^l)^The same as ^C^Br-∗-^D^Br. m) The *pure*-CS interaction of the HB nature with no covalency. ^n)^See ref. [39]. ^o)^The *regular*-CS interaction of the HB nature with no covalency.

## Data Availability

The data used to support the findings of this study are available in the supplementary information files.

## Supplementary Materials

Supplementary Materials Scheme S1: classification of interactions by the signs of ∇^2^*ρ*_b_(**r**_c_) and *H*_b_(**r**_c_), together with *G*_b_(**r**_c_) and *V*_b_(**r**_c_). Scheme S2: QTAIM-DFA: a plot of *H*_b_(**r**_c_) versus *H*_b_(**r**_c_) − *V*_b_(**r**_c_)/2 for weak to strong interactions. Scheme S3: rough classification and characterization of interactions by *θ* and *θ*_p_, together with *k*_b_(**r**_c_) (= *V*_b_(**r**_c_)/*G*_b_(**r**_c_)). QTAIM-DFA approach, computational data (Tables S2–S5 and Figures S3–S5), computation information and geometries of compounds, and graphical abstract. Figure S1: polar (*R*, *θ*) coordinate representation of *H*_b_(**r**_c_) versus *H*_b_(**r**_c_) − *V*_b_(**r**_c_)/2, with (*θ*_p_, *κ*_p_) parameters. Figure S2: plot of *H*_b_(**r**_c_) versus *w* in *r*(^1^Cl-^2^Cl) = *r*_o_(^1^Cl-^2^Cl) + *wa*_*o*_ for ^1^Cl-^2^Cl-^3^Cl^−^ (a) with the magnified picture of (a) (b) and that of *H*_b_(**r**_c_) − *V*_b_(**r**_c_)/2 versus *w* (c). Typical hydrogen bonds without covalency and typical hydrogen bonds with covalency are abbreviated as *t*-HB without cov. and *t*-HB with cov., respectively, whereas Cov-w and Cov-s stand for weak covalent bonds and strong covalent bonds, respectively. Table S1: proposed definitions for the classification and characterization of interactions.
