# Supplementary material for: Dynamic and Static Nature of Br4σ(4c–6e) and Se2Br5σ(7c–10e) in the Selenanthrene System and Related Species Elucidated by QTAIM Dual Functional Analysis with QC Calculations
Source: Bioinorg Chem Appl. 2020 Jul 24;2020:2901439. doi: 10.1155/2020/2901439 (PMC7396019; doi:10.1155/2020/2901439)
Supplement: Supplementary Materials — Scheme S1: classification of interactions by the signs of ∇2ρb(rc) and Hb(rc), together with Gb(rc) and Vb(rc). Scheme S2: QTAIM-DFA: a plot of Hb(rc) versus Hb(rc) − Vb(rc)/2 for weak to strong interactions. Scheme S3: rough classification and characterization of interactions by θ and θp, together with kb(rc) (= Vb(rc)/Gb(rc)). QTAIM-DFA approach, computational data (Tables S2–S5 and Figures S3–S5), computation information and geometries of compounds, and graphical abstract. Figure S1: polar (R, θ) coordinate representation of Hb(rc) versus Hb(rc) − Vb(rc)/2, with (θp, κp) parameters. Figure S2: plot of Hb(rc) versus w in r(1Cl-2Cl) = ro(1Cl-2Cl) + wao for 1Cl-2Cl-3Cl− (a) with the magnified picture of (a) (b) and that of Hb(rc) − Vb(rc)/2 versus w (c). Typical hydrogen bonds without covalency and typical hydrogen bonds with covalency are abbreviated as t-HB without cov. and t-HB with cov., respectively, whereas Cov-w and Cov-s stand for weak covalent bonds and strong covalent bonds, respectively. Table S1: proposed definitions for the classification and characterization of interactions. [file 2901439.f1.docx]

Supporting Information

**Dynamic and Static Nature of Br_4_ σ(4c–6e) and Se_2_Br_5_ σ(7c–10e) in Selenanthrene System and Related Species, Elucidated by QTAIM Dual Functional Analysis with QC Calculations**

Satoko Hayashi,* Taro Nishide, and Waro Nakanishi*

*Faculty of Systems Engineering, Wakayama University, 930 Sakaedani, Wakayama 640-8510, Japan. Fax: +81 73 457 8253; Tel: +81 73 457 8252; E-mail: hayashi3@sys.wakayama-u.ac.jp and nakanisi@sys.wakayama-u.ac.jp.*

| **Table of Contents** | **Pages** |
| --- | --- |
| Survey of QTAIM, closely related to QTAIM dual functional analysis | S2–S4 |
| Criteria for classification of interactions: behavior of typical interactions elucidated by QTAIM-DFA | S5 |
| Characterization of interactions | S5–S7 |
| Additional figures and tables | S8–S12 |
| References | S12 |
| Computation information and geometries of compounds | S13–S18 |

**Survey of QTAIM, closely related to QTAIM dual functional analysis (QTAIM-DFA)**

The bond critical point (BCP; *) is an important concept in QTAIM. The BCP of (*ω*, *σ*) = (3, –1)^S1^ is a point along the bond path (BP) at the interatomic surface, where charge density *ρ*(***r***) reaches a minimum. It is donated by *ρ*_b_(***r***_c_), so are other QTAIM functions, such as the total electron energy densities *H*_b_(***r***_c_), potential energy densities *V*_b_(***r***_c_), and kinetic energy densities *G*_b_(***r***_c_) at the BCPs. A chemical bond or interaction between A and B is denoted by A–B, which corresponds to the BP between A and B in QTAIM. We will use A-*-B for BP, where the asterisk emphasizes the presence of a BCP in A–B.

The sign of the Laplacian *ρ*_b_(***r***_c_) (∇^2^*ρ*_b_(***r***_c_)) indicates that *ρ*_b_(***r***_c_) is depleted or concentrated with respect to its surrounding, since ∇^2^*ρ*_b_(***r***_c_) is the second derivative of *ρ*_b_(***r***_c_). *ρ*_b_(***r***_c_) is locally depleted relative to the average distribution around ***r***_c_ if ∇^2^*ρ*_b_(***r***_c_) > 0, but it is concentrated when ∇^2^*ρ*_b_(***r***_c_) < 0. Total electron energy densities at BCPs (*H*_b_(***r***_c_)) must be a more appropriate measure for weak interactions on the energy basis.^S1–S8^ *H*_b_(***r***_c_) are the sum of kinetic energy densities (*G*_b_(***r***_c_)) and potential energy densities (*V*_b_(***r***_c_)) at BCPs, as shown in Equation (2) (S1). Electrons at BCPs are stabilized when *H*_b_(***r***_c_) < 0, therefore, interactions exhibit the covalent nature in this region, whereas they exhibit no covalency if *H*_b_(***r***_c_) > 0, due to the destabilization of electrons at BCPs under the conditions.^S1^ Equation (S2) represents the relation between ∇^2^*ρ*_b_(***r***_c_) and *H*_b_(***r***_c_), together with *G*_b_(***r***_c_) and *V*_b_(***r***_c_), which is closely related to the virial theorem.

*H*_b_(***r***_c_) = *G*_b_(***r***_c_) + *V*_b_(***r***_c_) (S1)

(*ћ*^2^/8*m*)∇^2^*ρ*_b_(***r***_c_) = *H*_b_(***r***_c_) – *V*_b_(***r***_c_)/2 (S2)

= *G*_b_(***r***_c_) + *V*_b_(***r***_c_)/2 (S2')

Interactions are classified by the signs of ∇^2^*ρ*_b_(***r***_c_) and *H*_b_(***r***_c_). Interactions in the region of ∇^2^*ρ*_b_(***r***_c_) < 0 are called shared-shell (SS) interactions and they are closed-shell (CS) interactions for ∇^2^*ρ*_b_(***r***_c_) > 0. *H*_b_(***r***_c_) must be negative when ∇^2^*ρ*_b_(***r***_c_) < 0, since *H*_b_(***r***_c_) are larger than (*ћ*^2^/8*m*)∇^2^*ρ*_b_(***r***_c_) by *V*_b_(***r***_c_)/2 with negative *V*_b_(***r***_c_) at all BCPs (Equation (S2)). Consequently, ∇^2^*ρ*_b_(***r***_c_) < 0 and *H*_b_(***r***_c_) < 0 for the SS interactions. The CS interactions are especially called *pure* CS interactions for *H*_b_(***r***_c_) > 0 and ∇^2^*ρ*_b_(***r***_c_) > 0, since electrons at BCPs are depleted and destabilized under the conditions.^S1a^ Electrons in the intermediate region between SS and *pure* CS, which belong to CS, are locally depleted but stabilized at BCPs, since ∇^2^*ρ*_b_(***r***_c_) > 0 but *H*_b_(***r***_c_) < 0.^S1a^ We call the interactions in this region *regular* CS,^S4,S5^ when it is necessary to distinguish from *pure* CS. The role of ∇^2^*ρ*_b_(***r***_c_) in the classification can be replaced by *H*_b_(***r***_c_) – *V*_b_(***r***_c_)/2, since (*ћ*^2^/8*m*)∇^2^*ρ*_b_(***r***_c_) = *H*_b_(***r***_c_) – *V*_b_(***r***_c_)/2 (Equation (S2)). Scheme S1 summarizes the classification.

**Scheme S1.** Classification of interactions by the signs of ∇^2^*ρ*_b_(***r***_c_) and *H*_b_(***r***_c_), together with *G*_b_(***r***_c_) and *V*_b_(***r***_c_).

We proposed QTAIM-DFA by plotting *H*_b_(***r***_c_) versus *H*_b_(***r***_c_) – *V*_b_(***r***_c_)/2 (= (*ћ*^2^/8*m*)∇^2^*ρ*_b_(***r***_c_)),^S4a^ after the proposal of *H*_b_(***r***_c_) versus ∇^2^*ρ*_b_(***r***_c_).^S4b^ Both axes in the plot of the former are given in energy unit, therefore, distances on the (*x*, *y*) (= (*H*_b_(***r***_c_) – *V*_b_(***r***_c_)/2, *H*_b_(***r***_c_)) plane can be expressed in the energy unit, which provides an analytical development. QTAIM-DFA incorporates the classification of interactions by the signs of ∇^2^*ρ*_b_(***r***_c_) and *H*_b_(***r***_c_). Scheme S2 summarizes the QTAIM-DFA treatment. Interactions of *pure* CS appear in the first quadrant, those of *regular* CS in the fourth quadrant and SS interactions do in the third quadrant. No interactions appear in the second one.

**Scheme S2.** QTAIM-DFA: Plot of *H*_b_(***r***_c_) versus *H*_b_(***r***_c_) – *V*_b_(***r***_c_)/2 for Weak to Strong Interactions

In our treatment, data for perturbed structures around fully optimized structures are also employed for the plots, together with the fully optimized ones (see Figure S1).^S4–S8^ We proposed the concept of the "dynamic nature of interaction" originated from the perturbed structures. The behavior of interactions at the fully optimized structures corresponds to "the static nature of interactions", whereas that containing perturbed structures exhibit the "dynamic nature of interaction" as explained below. The method to generate the perturbed structures is discussed later. Plots of *H*_b_(***r***_c_) versus *H*_b_(***r***_c_) – *V*_b_(***r***_c_)/2 are analyzed employing the polar coordinate (*R*, *θ*) representation with (*θ*_p_, *κ*_p_) parameters.^S4a,S5–S8^ Figure S1 explains the treatment. *R* in (*R*, *θ*) is defined by Equation (S3) and given in the energy unit. *R* corresponds to the energy for an interaction at BCP. The plots show a spiral stream, as a whole. *θ* in (*R*, *θ*) defined by Equation (S4), measured from the *y*-axis, controls the spiral stream of the plot. Each plot for an interaction shows a specific curve, which provides important information of the interaction (see Figure S1). The curve is expressed by *θ*_p_ and *κ*_p_. While *θ*_p_, defined by Equation (S5) and measured from the *y*-direction, corresponds to the tangent line of a plot, where *θ*_p_ is calculated employing data of the perturbed structures with a fully-optimized structure and *κ*_p_ is the curvature of the plot (Equation (S6)). While (*R*, *θ*) correspond to the static nature, (*θ*_p_, *κ*_p_) represent the dynamic nature of interactions. We call (*R*, *θ*) and (*θ*_p_, *κ*_p_) QTAIM-DFA parameters, whereas *ρ*_b_(***r***_c_), ∇^2^*ρ*_b_(***r***_c_), *G*_b_(***r***_c_), *V*_b_(***r***_c_), *H*_b_(***r***_c_) and *H*_b_(***r***_c_) – *V*_b_(***r***_c_)/2 belong to QTAIM functions. *k*_b_(***r***_c_), defined by Equation (S7), is an QTAIM function but it will be treated as if it were an QTAIM-DFA parameter, if suitable.

**Figure S1.** Polar (*R*, *θ*) coordinate representation of *H_b_*(***r***_c_) versus *H_b_*(***r***_c_) – *V_b_*(***r***_c_)/2, with (*θ*_p_, *κ*_p_) parameters.

*R* = (*x*^2^ + *y*^2^)^1/2^ (S3)

*θ* = 90º – tan^–1^ (*y*/*x*) (S4)

*θ*_p_ = 90º – tan^–1^ (d*y*/d*x*) (S5)

*κ*_p_ = ⎜d^2^*y*/d*x*^2^⎜/[1 + (d*y*/d*x*)^2^]^3/2^ (S6)

*k*_b_(***r***_c_) = *V*_b_(***r***_c_)/*G*_b_(***r***_c_) (S7)

where (*x*, *y*) = (*H*_b_(***r***_c_) – *V*_b_(***r***_c_)/2, *H*_b_(***r***_c_))

**Criteria for classification of interactions: behavior of typical interactions elucidated by QTAIM-DFA**

*H*_b_(***r***_c_) are plotted versus *H*_b_(***r***_c_) – *V*_b_(***r***_c_)/2 for typical interactions in vdW (van der Waals interactions), HBs (hydrogen bonds), CT-MCs (molecular complexes through charge transfer), X_3_^–^ (trihalide ions), CT-TBPs (trigonal bipyramidal adducts through charge-transfer), Cov-w (weak covalent bonds) and Cov-s (strong covalent bonds).^S4–S8^ Rough criteria are obtained by applying QTAIM-DFA, after the analysis of the plots for the typical interactions according to Equations (S3)–(S7). Scheme S3 shows the rough criteria, which are accomplished by the *θ* and *θ*_p_ values, together with the values of *k*_b_(***r***_c_). The criteria will be employed to discuss the nature of interactions in question, as a reference.

**
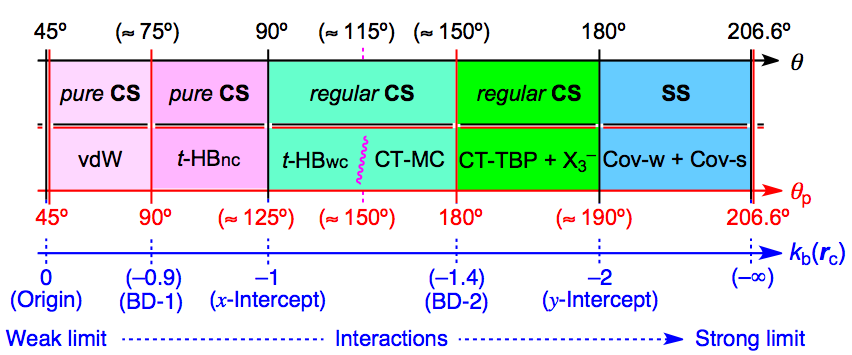
**

**Scheme S3.** Rough classification and characterization of interactions by *θ* and *θ*_p_, together with *k*_b_(***r***_c_) (= *V*_b_(***r***_c_)/*G*_b_(***r***_c_)).

**Characterization of interactions**

The characterization of interactions is explained employing [^1^Cl-^2^Cl-^3^Cl]^–^. The wide range of the perturbed structures were generated by partially optimizing *r*(^2^Cl-^3^Cl) in [^1^Cl-^2^Cl-^3^Cl]^–^, assuming the *C*_∞v_ symmetry, with *r*(^1^Cl-^2^Cl) being fixed in the wide range. The partial optimization method is called POM.^S4b,S5^ The QTAIM functions, such as *V*_b_(***r***_c_), *G*_b_(***r***_c_), *H*_b_(***r***_c_), *H*_b_(***r***_c_) – *V*_b_(***r***_c_)/2 are calculated at BCPs for the wide varieties of the perturbed structures of [^1^Cl-^2^Cl-^3^Cl]^–^. *H*_b_(***r***_c_) – *V*_b_(***r***_c_)/2 and *H*_b_(***r***_c_) are plotted versus the interaction distances *r*(^1^Cl-^2^Cl) in the perturbed structures of [^1^Cl-^2^Cl-^3^Cl]^–^, in the wide range. Figure S2 shows the plots. Each plot is analyzed using a regression curve of the ninth function and the first derivative of each regression curve is obtained. As shown in Figure S2, the maximum value of *H*_b_(***r***_c_) (d(*H*_b_(***r***_c_)/d*r* = 0) is defined as the borderline between vdW and t-HB interactions. Similarly, the maximum value of *H*_b_(***r***_c_) – *V*_b_(***r***_c_)/2 (d(*H*_b_(***r***_c_) – *V*_b_(***r***_c_)/2)/d*r* = 0) does to the borderline between CT-MC and CT-TBP. However, it seems difficult to find a characteristic point corresponding to the borderline between *t*-HB and CT-MC in nature. Therefore, the borderline is tentatively given by *θ*_p_ = 150º based on the expectation form the experimental results, where *θ*_p_ is defined by [90º – tan^–1^[d*H*_b_(***r***_c_)/d(*H*_b_(***r***_c_) – *V*_b_(***r***_c_)/2)]] in the plot of *H*_b_(***r***_c_) versus *H*_b_(***r***_c_) – *V*_b_(***r***_c_)/2. The proposed classification and characterization of interactions, by means of the QTAIM functions of *H*_b_(***r***_c_), *H*_b_(***r***_c_) – *V*_b_(***r***_c_)/2, *G*_b_(***r***_c_), and/or *V*_b_(***r***_c_), are summarized in Table S1.


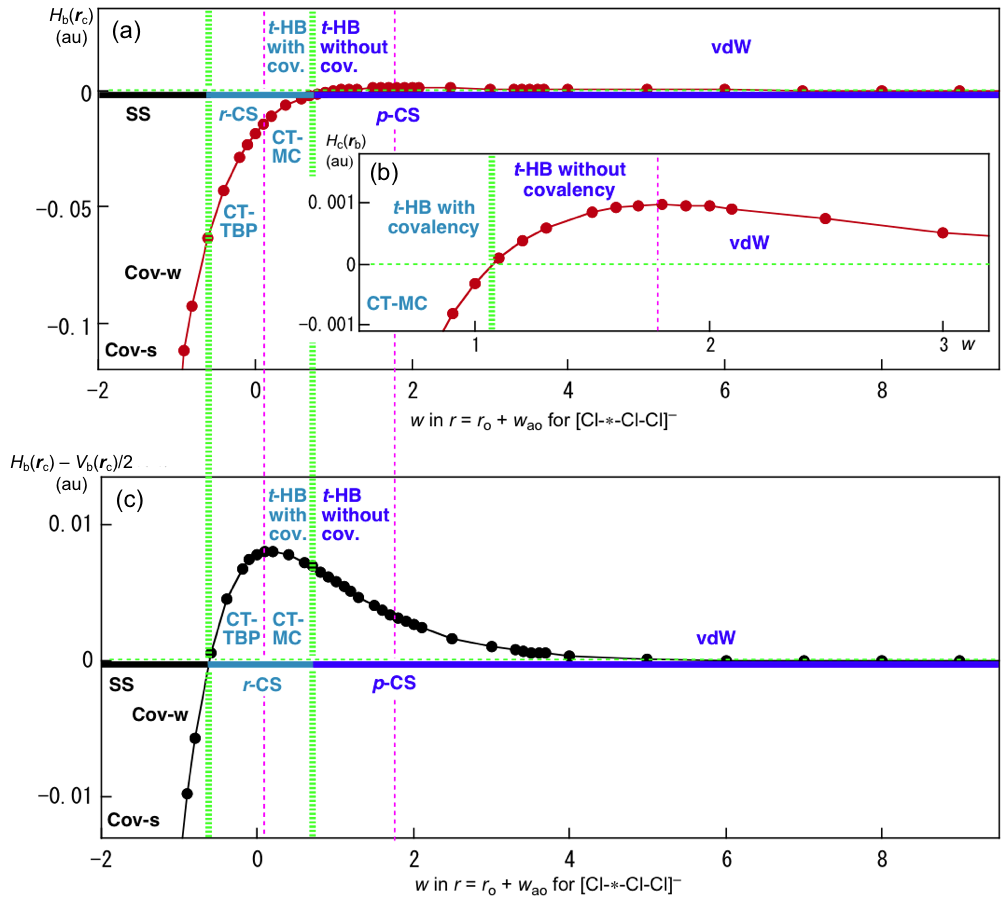


**Figure S2.** Plot of *H*_b_(***r***_c_) versus *w* in *r*(^1^Cl-^2^Cl) = *r*_o_(^1^Cl-^2^Cl) + *wa*_o_ for ^1^Cl-^2^Cl-^3^Cl^–^ (a) with the magnified picture of (a) (b) and that of *H*_b_(***r***_c_) – *V*_b_(***r***_c_)/2 versus *w* (c). Typical hydrogen bonds without covalency and typical hydrogen bonds with covalency are abbreviated as *t*-HB without cov. and *t*-HB with cov., respectively, whereas Cov-w and Cov-s stand for weak covalent bonds and strong covalent bonds, respectively.

**Table S1.** Proposed definitions for the classification and characterization of interactions by the signs *H*_b_(***r***_c_) and *H*_b_(***r***_c_) – *V*_b_(***r***_c_)/2 and their first derivatives, together with the tentatively proposed definitions by the characteristic points on the plots of *H*_b_(***r***_c_) versus *H*_b_(***r***_c_) – *V*_b_(***r***_c_)/2. The tentatively proposed definitions are shown by *italic*. The requirements for the interactions are also shown.

ChP/Interaction Requirements by *H*_b_(***r***_c_) and *V*_b_(***r***_c_) Requirements by *G*_b_(***r***_c_) and *V*_b_(***r***_c_)

Origin *H*_b_(***r***_c_) – *V*_b_(***r***_c_)/2 = 0; *H*_b_(***r***_c_) = 0 *G*_b_(***r***_c_) = 0; *V*_b_(***r***_c_) = 0

vdW *H*_b_(***r***_c_) > 0; d*H*_b_(***r***_c_)/d(–*r*) > 0 *G*_b_(***r***_c_) > –*V*_b_(***r***_c_); d*G*_b_(***r***_c_)/d(–*r*) > –d*V*_b_(***r***_c_)/d(–*r*)

Borderline (BD-1) *H*_b_(***r***_c_) > 0; d*H*_b_(***r***_c_)/d(–*r*) = 0 *G*_b_(***r***_c_) > –*V*_b_(***r***_c_); d*G*_b_(***r***_c_)/d(–*r*) = –d*V*_b_(***r***_c_)/d(–*r*)

*t*-HB_with no covalency_ *H*_b_(***r***_c_) > 0; d*H*_b_(***r***_c_)/d(–*r*) < 0 *G*_b_(***r***_c_) > –*V*_b_(***r***_c_); d*G*_b_(***r***_c_) < –d*V*_b_(***r***_c_)

Borderline (*x*-intercept) *H*_b_(***r***_c_) = 0 (*θ*_p_^a)^ = 125º) *G*_b_(***r***_c_) = –*V*_b_(***r***_c_) (*θ*_p_^a)^ = 125º)

*t*-HB_with covalency_ *H*_b_(***r***_c_) < 0; (125º <) *θ*_p_^a)^ < 150º *G*_b_(***r***_c_) < –*V*_b_(***r***_c_); (125º <) *θ*_p_^b)^ < 150º

*Borderline (Tentative) θ*_p_^a)^ *= 150º θ*_p_^b)^ *= 150º*

CT-MC d(*H*_b_(***r***_c_) – *V*_b_(***r***_c_)/2)/d(–*r*) > 0; d*G*_b_(***r***_c_) > d*V*_b_(***r***_c_)/2;

150º < *θ*_p_^a)^ < 180º 150º < *θ*_p_^a)^ < 180º

Borderline (BD-2) d(*H*_b_(***r***_c_) – *V*_b_(***r***_c_)/2)/d(–*r*) = 0 2d*G*_b_(***r***_c_)/d(–*r*) = –d*V*_b_(***r***_c_)/d(–*r*)

(*H*_b_(***r***_c_) – *V*_b_(***r***_c_)/2 > 0; *H*_b_(***r***_c_) < 0) (–*V*_b_(***r***_c_)/2 < *G*_b_(***r***_c_) < –*V*_b_(***r***_c_))

CT-TBP with X_3_^–^ d(*H*_b_(***r***_c_) – *V*_b_(***r***_c_)/2)/d(–*r*) < 0 2d*G*_b_(***r***_c_)/d(–*r*) < –d*V*_b_(***r***_c_)/d(–*r*)

(*H*_b_(***r***_c_) – *V*_b_(***r***_c_)/2 > 0; *H*_b_(***r***_c_) < 0) (–*V*_b_(***r***_c_)/2 < *G*_b_(***r***_c_) < –*V*_b_(***r***_c_))

Borderline (*y*-intercept) *H*_b_(***r***_c_) – *V*_b_(***r***_c_)/2 = 0 (*H*_b_(***r***_c_) < 0) *G*_b_(***r***_c_) = –*V*_b_(***r***_c_)/2 (*G*_b_(***r***_c_) < –*V*_b_(***r***_c_))

Cov-w *H*_b_(***r***_c_) – *V*_b_(***r***_c_)/2 < 0; *R*^c)^ *< 0.15 au* *G*_b_(***r***_c_) < –*V*_b_(***r***_c_)/2; *R*^c)^ *< 0.15 au*

*Borderline (Tentative) R*^c)^ *= 0.15 au R*^d)^ *= 0.15 au*

Cov-s *H*_b_(***r***_c_) – *V*_b_(***r***_c_)/2 < 0; *R*^c)^ *> 0.15 au* *G*_b_(***r***_c_) < –*V*_b_(***r***_c_)/2; *R*^d)^ *> 0.15 au*

a) *θ*_p_ = 90º – tan^–1^ [d*H*_b_(***r***_c_)/d(*H*_b_(***r***_c_) – *V*_b_(***r***_c_)/2)], *θ*_p_ = 125º is tentatively given for *θ* = 90º, where *θ* is defined by 90º – tan^–1^[*H*_b_(***r***_c_)/(*H*_b_(***r***_c_) – *V*_b_(***r***_c_)/2)] with *H*_b_(***r***_c_) = 0. b) *θ*_p_ = 90º – tan^–1^[d(*G*_b_(***r***_c_) + *V*_b_(***r***_c_))/d(*G*_b_(***r***_c_) + *V*_b_(***r***_c_)/2)], *θ*_p_ = 125º is tentatively given for *θ* = 90º, where *θ* is defined by 90º – tan^–1^[(*G*_b_(***r***_c_) + *V*_b_(***r***_c_))/(*G*_b_(***r***_c_) + *V*_b_(***r***_c_)/2)] with (*G*_b_(***r***_c_) + *V*_b_(***r***_c_)) = 0]. c) *R* = [(*H*_b_(***r***_c_) – *V*_b_(***r***_c_)/2)^2^ + (*H*_b_(***r***_c_))^2^]^1/2^. d) *R* = [(*G*_b_(***r***_c_) + *V*_b_(***r***_c_)/2)^2^ + (*G*_b_(***r***_c_) + *V*_b_(***r***_c_))^2^]^1/2^.

**Table S2.** Structural parameters, Δ*E*_ES_, and Δ*E*_ZP_ for ^B^Br-*-^A^Br-*-^A^Br-*-^B^Br at BCPs in Br_4_ σ(4c–6e), together with ^A^Br-*-^A^Br in Br_2_, evaluated with MP2/BSS-A^a)^

Compd *r*(^A^Br, ^A^Br) *r*(^A^Br, ^B^Br) Δ*r*(^A^Br, ^B^Br) ∠R^A^Br^B^Br ∠^A^Br^A^Br^B^Br Δ*E*_ES_ Δ*E*_ZP_

(symm) (Å) (Å) (Å) (°) (°) (kJ mol^-1^) (kJ mol^-1^)

Br_2_ (*D*_∞h_) 2.2756

Br_4_^2–^ (D_∞h_) 2.3874 3.1698 -0.5302 75.0 76.1

Br_4_^2–^ (D_∞h_)_obsd_ 2.387(2) 2.973(2) -0.727

Br_6_ (*C*_2_)^b)^ 2.2910 3.3277 -0.3723 89.55 176.44 -23.4 -21.9

Br_6_ (*C*_2h_) 2.2910 3.3277 -0.3723 89.60 176.49 -23.4 -21.9

Br_6_ (*C*_2h_)_obsd_ 2.4906 3.2509 -0.4491 101.30 169.92

H_2_Br_4_ (*C*_2h_) 2.2909 3.3814 -0.3186 90.57 177.83 -19.9 -17.0

Me_2_Br_4_ (*C*_2h_) 2.3034 3.2336 -0.4664 83.90 172.75 -33.3 -30.2

H_4_Se_2_Br_6_ (*C*_i_) 2.3281 3.1093 -0.5907 81.94 169.79 -58.7 -56.3

Me_4_Se_2_Br_6_ (*C*_i_) 2.3270 3.1224 -0.5776 74.63 171.66 -61.7 -58.5

**5** (*C*_i_) 2.3331 3.0937 -0.6063 73.81 171.17 -70.4 -68.2

**6** (*C*_i_) 2.3343 3.0916 -0.6084 73.56 171.25 -70.4 -68.1

**1** (*C*_i_)_obsd_ 2.3311 3.1338 -0.5663 102.60 172.02

**1** (*C*_i_)_CAM_^b)^ 2.3169 3.3661 -0.3339

a) See the text for BSS. b) ∠ ^C^Br^B^Br^A^Br^A’^Br = 72.7°, ∠ ^C’^Br^B’^Br^A’^Br^A^Br = 75.0°. b) CAM-B3LYP level.

**Table S3.** Structural parameters, Δ*E*_ES_, and Δ*E*_ZP_ for ^A^Br-*-^A^Se-*-^B^Br-*-^C^Br-*-^D^Br-*-^B^Se-*-^E^Br at BCPs in **7** (*C*_i_), **8** (*C*_i_), and **2** (*C*_1_)_obsd_, together with ^A^Br-*-^A^Se-*-^B^Br in **3** (*C*_s_) and ^A^Br-*-^A^Se-*-^B^Br-*-^C^Br-*-^D^Br in **4** (*C*_s_), evaluated with MP2/BSS-A^a)^

Compd *r*(^A^Se, ^A^Br) *r*(^A^Se, ^B^Br) *r*(^B^Br, ^C^Br) *r*(^C^Br, ^D^Br) *r*(^D^Br, ^B^Se) *r*(^B^Se, ^E^Br)

(symm) (Å) (Å) (Å) (Å) (Å) (Å)

**7** (*C*_i_) 2.8197 2.4687 2.9005 2.9005 2.4687 2.8197

**8** (*C*_i_) 2.7397 2.5795 2.8194 2.8194 2.5795 2.7397

**2** (*C*_1_)_obsd_ 3.1962 2.6413 2.3614 3.1608 2.4470 2.4938

**3** (*C*_s_) 2.5102 2.5602

**4** (*C*_s_) 3.4895 2.8095 2.3741

(continued)

Compd Δ*r*(^A^Se, ^A^Br) Δ*r*(^A^Se, ^B^Br) Δ*r*(^B^Br, ^C^Br) Δ*r*(^C^Br, ^D^Br) Δ*r*(^D^Br, ^B^Se) Δ*r*(^B^Se, ^E^Br)

(symm) (Å) (Å) (Å) (Å) (Å) (Å)

**7** (*C*_i_) -0.9303 -1.2813 -0.7995 -0.7995 -1.2813 -0.9303

**8** (*C*_i_) -1.0103 -1.1705 -0.8806 -0.8806 -1.1705 -1.0103

**2** (*C*_1_)_obsd_ -0.5538 -1.1087 -1.3386 -0.5392 -1.3030 -1.2562

**3** (*C*_s_) -1.2398 -1.1898

**4** (*C*_s_) -0.2605 -0.9405 -1.3250

(continued)

Compd Δ*E*_ES_ Δ*E*_ZP_

(symm) (kJ mol^-1^) (kJ mol^-1^)

**7** (*C*_i_) -75.3 -75.7

**8** (*C*_i_) -63.5 -64.6

**2** (*C*_1_)_obsd_

**3** (*C*_s_)

**4** (*C*_s_) -42.7 -41.0

a) See BSS in the text.

**Table S4.** S NBO analysis for Br_4_ σ(4c–6e) of the n_p_(^B^Br)→σ*(^A^Br–^A^Br)←n_p_(^B^Br) type, evaluated with MP2/BSS-A^a,b)^

Species *E*_2_ (*ε_j_* – *ε_i_*) *F*(*i,j*)

(symmetry) (kJ mol^–1^) (au) (au)

Br_4_^2–^ (*D*_∞h_)^c)^ 59.71 0.43 0.071

Br_6_ (*C*_2_) 20.71 0.58 0.048

Br_6_ (*C*_2h_)^d)^ 20.71 0.58 0.048

Br_6_ (*C*_2h_)_obsd_ 30.25 0.52 0.055

H_2_Br_4_ (*C*_2h_) 19.24 0.58 0.046

Me_2_Br_4_ (*C*_2h_) 30.59 0.53 0.056

H_4_Se_2_Br_6_ (*C*_i_) 52.55 0.49 0.071

Me_4_Se_2_Br_6_ (*C*_i_) 46.69 0.49 0.066

**5** (*C*_i_)^e)^ 50.88 0.49 0.069

**6** (*C*_i_) 51.38 0.48 0.069

**1** (*C*_i_)_obsd_ 44.48 0.52 0.067

a) See BSS in the text. b) Only one side of the CT interaction is shown. c) With one imaginary frequency for the vibration mode of the SGU symmetry. d) With one imaginary frequency for the rotational mode around the linear Br_4_ interaction. e) With one imaginary frequency for the vibration mode of the AU symmetry.

**Table S5.** The *r*_BP_ and *R*_SL_ values evaluated with MP2/BSS-A for the optimized and observed structures of R'Br_4_R', together with the Δ*r*_BP_ values^a)^

species X-*-Y *r*_BP_^b)^ *R*_SL_^c)^ Δ*r*_BP_^d)^

(symm) (Å) (Å) (Å)

Br_2_ (*D*_∞h_) ^A^Br-*-^A^Br 2.2756 2.2756 0.0000

Br_4_^2–^ (*D*_∞h_)^e)^ ^A^Br-*-^A^Br 2.3874 2.3874 0.0000

^A^Br-*-^B^Br 3.1698 3.1698 0.0000

Br_6_ (*C*_2_) ^A^Br-*-^A^Br 2.2910 2.2910 0.0000

^A^Br-*-^B^Br 3.3283 3.3277 0.0006

Br_6_ (*C*_2h_)^f)^ ^A^Br-*-^A^Br 2.2911 2.2910 0.0000

^A^Br-*-^B^Br 3.3282 3.3277 0.0006

Br_6_ (*C*_2h_)_obsd_ ^A^Br-*-^A^Br 2.4906 2.4906 0.0000

^A^Br-*-^B^Br 3.2531 3.2509 0.0021

H_2_Br_4_ (*C*_2h_) ^A^Br-*-^A^Br 2.2909 2.2909 0.0000

^A^Br-*-^B^Br 3.3820 3.3814 0.0006

Me_2_Br_4_ (*C*_2h_) ^A^Br-*-^A^Br 2.3034 2.3034 0.0000

^A^Br-*-^B^Br 3.2354 3.2336 0.0018

H_4_Se_2_Br_6_ (*C*_i_) ^A^Br-*-^A^Br 2.3281 2.3281 0.0000

^A^Br-*-^B^Br 3.1124 3.1093 0.0031

Me_4_Se_2_Br_6_ (*C*_i_) ^A^Br-*-^A^Br 2.3270 2.3270 0.0000

^A^Br-*-^B^Br 3.1251 3.1224 0.0027

**5** (*C*_i_)^g)^ ^A^Br-*-^A^Br 2.3332 2.3331 0.0000

^A^Br-*-^B^Br 3.0963 3.0937 0.0026

**6** (*C*_i_) ^A^Br-*-^A^Br 2.3343 2.3343 0.0000

^A^Br-*-^B^Br 3.0942 3.0916 0.0026

**1** (*C*_i_)_obsd_ ^A^Br-*-^A^Br 2.3311 2.3311 0.0000

^A^Br-*-^B^Br 3.1346 3.1338 0.0008

**7** (*C*_i_) ^A^Br-*-^B^Br 2.8965 2.8965 0.0001

^B^Br-*-^C^Br 2.4700 2.4691 0.0009

^C^Br-*-^D^Br 2.8207 2.8177 0.0031

**8** (*C*_i_) ^A^Br-*-^B^Br 2.8133 2.8133 0.0000

^B^Br-*-^C^Br 2.5832 2.5821 0.0011

^C^Br-*-^D^Br 2.7411 2.7397 0.0014

**2** (*C*_1_)_obsd_ ^A^Br-*-^B^Br 3.1978 3.1962 0.0016

^B^Br-*-^C^Br 2.6421 2.6413 0.0008

^C^Br-*-^D^Br 2.3614 2.3614 0.0000

^D^Br-*-^E^Br 3.1632 3.1608 0.0025

^E^Br-*-^F^Br 2.4487 2.4470 0.0017

^F^Br-*-^G^Br 2.4951 2.4938 0.0013

**3** (*C*_s_) ^A^Br-*-^B^Br 2.5113 2.5102 0.0011

^A^Br-*-^B'^Br 2.5618 2.5602 0.0016

**4** (*C*_s_) ^A^Br-*-^B^Br 3.4920 3.4895 0.0025

^B^Br-*-^C^Br 2.8098 2.8095 0.0003

^C^Br-*-^D^Br 2.3741 2.3741 0.0000

a) See text for BSS-A. b) The lengths of BPs. c) Straight-line distances. d) Δ*r*_BP_ = *r*_BP_ – *R*_SL_. e) With one imaginary frequency for the vibration mode of the SGU symmetry. f) With one imaginary frequency for the rotational mode around the linear Br_4_ interaction. g) With one imaginary frequency for the vibration mode of the AU symmetry.


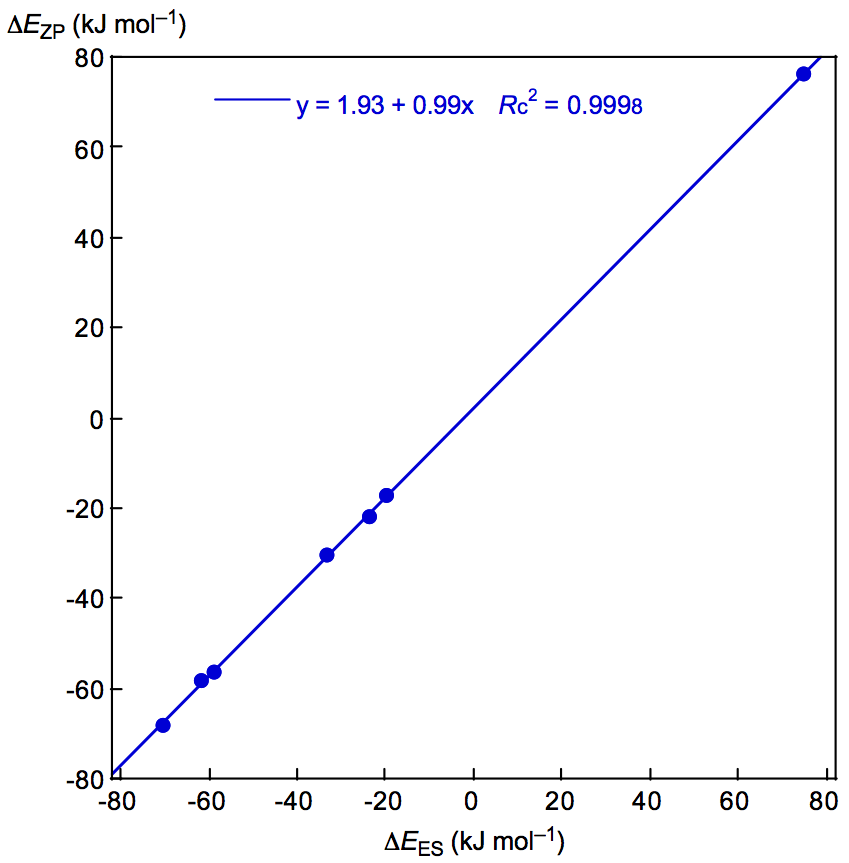


**Figure S3.** Plot of Δ*E*_ZP_ versus Δ*E*_ES_ for the optimized structures of R'–^B^Br---^A^Br–^A^Br---^B^Br–R', evaluated with MP2/BSS-A


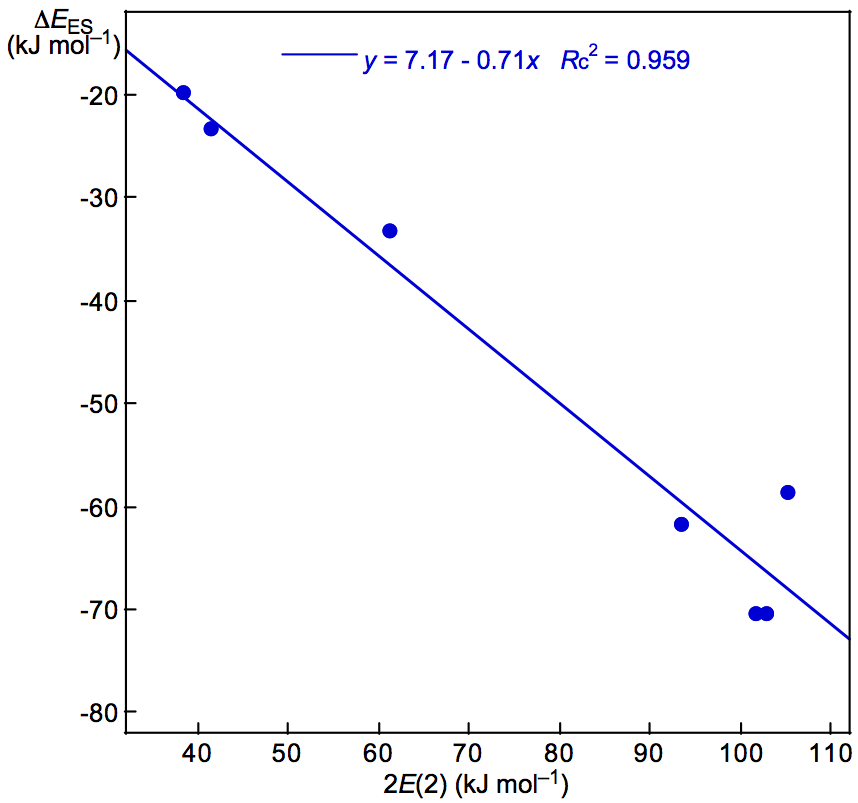


**Figure S4.** Plots of Δ*E*_ES_ versus 2*E*(2) for the optimized structures of R'–^B^Br---^A^Br–^A^Br---^B^Br–R', except for Br_4_^2–^ (*D*_∞h_), evaluated with MP2/BSS-A


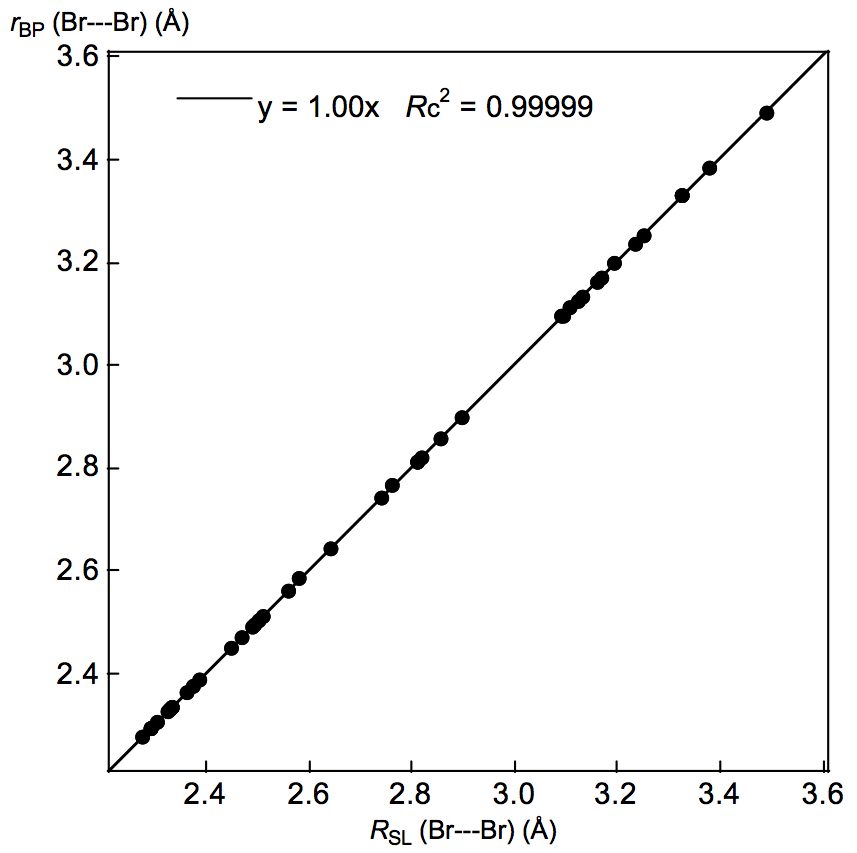


**Figure S5.** Plot of *r*_BP_ (^A^Br---^A^Br or ^A^Br-*-^B^Br) versus *R*_SL_ (^A^Br---^A^Br or ^A^Br-*-^B^Br) for the optimized structures of Br_4_ σ(4c–6e) with MP2/BSS-A

**References**

S1 (a) *Atoms in Molecules. A Quantum Theory*: eds. R. F. W. Bader, Oxford University Press, Oxford, UK, 1990; (b) C. F. Matta, R. J. Boyd, *An Introduction to the Quantum Theory of Atoms in Molecules* in *The Quantum Theory of Atoms in Molecules: From Solid State to DNA and Drug Design*: eds. C. F. Matta, R. J. Boyd, WILEY-VCH, Weinheim, Germany, 2007, Chapter 1.

S2 (a) R. F. W. Bader, T. S. Slee. D. Cremer, E. Kraka, *J. Am. Chem. Soc.* **1983**, *105*, 5061–5068; (b) R. F. W. Bader, *Chem. Rev.* **1991**, *91*, 893–926; (c) R. F. W. Bader, *J. Phys. Chem. A* **1998**, *102*, 7314–7323; (d) F. Biegler-König, R. F. W. Bader, T. H. Tang, *J. Comput. Chem.* **1982**, ***3***, 317–328; (e) R. F. W. Bader, *Acc. Chem. Res.* **1985**, *18*, 9–15; (f) T. H. Tang, R. F. W. Bader, P. MacDougall, *Inorg. Chem.* **1985**, *24*, 2047–2053; (g) F. Biegler-König, J. Schönbohm, D. Bayles, *J. Comput. Chem*. **2001**, *22*, 545–559; (h) F. Biegler-König, J. Schönbohm, *J. Comput. Chem.* **2002**, *23*, 1489–1494.

S3 W. Nakanishi, T. Nakamoto, S. Hayashi, T. Sasamori, N. Tokitoh, *Chem. Eur. J.* **2007**, *13*, 255–268.

S4 (a) W. Nakanishi, S. Hayashi, K. Narahara, *J. Phys. Chem. A* **2009**, *113*, 10050–10057; (*b*) W. Nakanishi, S. Hayashi, K. Narahara, *J. Phys. Chem. A* **2008**, *112*, 13593–13599.

S5 W. Nakanishi, S. Hayashi, *Curr. Org. Chem.* **2010**, *14*, 181–197.

S6 (a) W. Nakanishi, S. Hayashi, *J. Phys. Chem. A* **2010**, *114*, 7423–7430; (b) W. Nakanishi, S. Hayashi, K. Matsuiwa, M. Kitamoto, *Bull. Chem. Soc. Jpn* **2012**, *85*, 1293–1305.

S7 W. Nakanishi, S. Hayashi, *J. Phys. Chem. A* **2013**, *117*, 1795–1803.

S8 W. Nakanishi, S. Hayashi, *Int. J. Quantum Chem.* **2018**, *118*, e25590.

**Optimized structures given by Cartesian coordinates**

Optimized structures given by Cartesian coordinates for examined molecules, together with the total energies with the MP2/BSS-A method of the Gaussian 09 program.

MP2/BSS-A

Adduct Br_2_

Symmetry *D*_∞h_

energy MP2 = –5145.248000 au

Standard orientation

35 0 0.000000 0.000000 1.137779

35 0 0.000000 0.000000 -1.137779

MP2/BSS-A

Adduct Br_4_^2–^

Symmetry *D*_∞h_

energy MP2 = –10290.633850 au

Standard orientation

35 0 0.000000 0.000000 4.363449

35 0 0.000000 0.000000 1.193674

35 0 0.000000 0.000000 -1.193674

35 0 0.000000 0.000000 -4.363449

MP2/BSS-A

Adduct Br_6_

Symmetry *C*_2_

energy MP2 = –15435.752929au

Standard orientation

35 0 0.692273 4.509122 1.257364

35 0 -0.692273 4.414577 -0.545566

35 0 -0.208369 1.126404 -0.711798

35 0 0.208369 -1.126404 -0.711798

35 0 0.692273 -4.414577 -0.545566

35 0 -0.692273 -4.509122 1.257364

MP2/BSS-A

Adduct Br_6_

Symmetry *C*_2h_

energy MP2 = –15435.752915 au

Standard orientation

35 0 -0.041534 1.144768 0.000000

35 0 0.041534 -1.144768 0.000000

35 0 -0.041534 -4.471410 0.000000

35 0 0.041534 4.471410 0.000000

35 0 -2.315541 -4.398540 0.000000

35 0 2.315541 4.398540 0.000000

MP2/BSS-A

Adduct H_2_Br_4_

Symmetry *C*_2h_

energy MP2 = –10291.700823 au

Standard orientation

1 0 -1.373916 4.540151 0.000000

35 0 0.043451 4.526074 0.000000

35 0 0.043451 1.144631 0.000000

35 0 -0.043451 -1.144631 0.000000

35 0 -0.043451 -4.526074 0.000000

1 0 1.373916 -4.540151 0.000000

MP2/BSS-A

Adduct Me_2_Br_4_

Symmetry *C*_2h_

energy MP2 = –10370.056498au

Standard orientation

1 0 1.339487 3.936468 0.901819

1 0 1.339487 3.936468 -0.901819

1 0 1.210931 5.497074 0.000000

6 0 0.965508 4.430578 0.000000

35 0 -0.965508 4.270725 0.000000

35 0 -0.357568 1.094793 0.000000

35 0 0.357568 -1.094793 0.000000

35 0 0.965508 -4.270725 0.000000

6 0 -0.965508 -4.430578 0.000000

1 0 -1.339487 -3.936468 0.901819

1 0 -1.339487 -3.936468 -0.901819

1 0 -1.210931 -5.497074 0.000000

MP2/BSS-A

Adduct H_4_Se_2_Br_6_

Symmetry *C*_i_

energy MP2 = –20238.059119 au

Standard orientation

35 0 -1.105687 -0.329185 0.155163

35 0 1.105687 0.329185 -0.155163

35 0 -4.173768 -0.668495 0.528990

35 0 4.173768 0.668495 -0.528990

34 0 -4.122126 1.873249 0.282521

1 0 -4.371889 1.631216 -1.146133

1 0 -2.664246 1.657627 0.171856

34 0 4.122126 -1.873249 -0.282521

1 0 4.371889 -1.631216 1.146133

1 0 2.664246 -1.657627 -0.171856

35 0 -3.766476 4.269742 -0.259597

35 0 3.766476 -4.269742 0.259597

MP2/BSS-A

Adduct Me_4_Se_2_Br_6_

Symmetry *C*_i_

energy MP2 = –20394.831995au

Standard orientation

35 0 -0.907656 -0.689249 0.234098

35 0 0.907656 0.689249 -0.234098

35 0 -3.601114 -2.185718 0.739180

35 0 3.601114 2.185718 -0.739180

34 0 -4.239414 0.288469 0.451441

34 0 4.239414 -0.288469 -0.451441

35 0 -4.867513 2.693312 0.218953

35 0 4.867513 -2.693312 -0.218953

6 0 -6.117247 -0.249896 0.463374

1 0 -6.396626 -0.370418 1.518443

1 0 -6.694676 0.551118 -0.011938

1 0 -6.188991 -1.206754 -0.066172

6 0 -3.948039 0.202723 -1.476810

1 0 -3.073016 0.832548 -1.679627

1 0 -4.842901 0.614856 -1.958750

1 0 -3.764148 -0.845545 -1.738432

6 0 3.948039 -0.202723 1.476810

1 0 3.073016 -0.832548 1.679627

1 0 4.842901 -0.614856 1.958750

1 0 3.764148 0.845545 1.738432

6 0 6.117247 0.249896 -0.463374

1 0 6.396626 0.370418 -1.518443

1 0 6.188991 1.206754 0.066172

1 0 6.694676 -0.551118 0.011938

MP2/BSS-A

Adduct **5**: C_8_H_8_Se_4_Br_6_

Symmetry *C*_i_

energy MP2 = –25344.242828 au

Standard orientation

35 0 -0.890693 -0.717287 0.230337

35 0 0.890693 0.717287 -0.230337

35 0 -3.531230 -2.248624 0.733791

35 0 3.531230 2.248624 -0.733791

34 0 -4.171760 0.215873 0.487887

34 0 4.171760 -0.215873 -0.487887

35 0 -4.768361 2.665946 0.306112

35 0 4.768361 -2.665946 -0.306112

6 0 -6.027568 -0.218353 0.463083

6 0 -3.807373 0.178184 -1.382250

6 0 -6.636402 -0.987769 -0.465061

6 0 -4.454834 -0.590790 -2.283241

1 0 -6.574762 0.252724 1.283414

1 0 -3.026733 0.888473 -1.663912

1 0 -7.706850 -1.189007 -0.353925

1 0 -4.137382 -0.540545 -3.329850

34 0 -5.911982 -1.747442 -2.022189

6 0 3.807373 -0.178184 1.382250

6 0 6.027568 0.218353 -0.463083

6 0 4.454834 0.590790 2.283241

6 0 6.636402 0.987769 0.465061

1 0 3.026733 -0.888473 1.663912

1 0 6.574762 -0.252724 -1.283414

1 0 4.137382 0.540545 3.329850

1 0 7.706850 1.189007 0.353925

34 0 5.911982 1.747442 2.022189

MP2/BSS-A

Adduct **6**: C_8_H_8_Se_2_S_2_Br_6_

Symmetry *C*_i_

energy MP2 = –21339.592649 au

Standard orientation

35 0 -0.890820 -0.717696 0.231568

35 0 0.890820 0.717696 -0.231568

35 0 -3.526610 -2.249222 0.746543

35 0 3.526610 2.249222 -0.746543

34 0 -4.161890 0.223538 0.489790

34 0 4.161890 -0.223538 -0.489790

35 0 -4.741290 2.677084 0.310777

35 0 4.741290 -2.677084 -0.310777

6 0 -6.009967 -0.219924 0.446308

6 0 -3.823110 0.155533 -1.378930

6 0 -6.594802 -0.969576 -0.515669

6 0 -4.520446 -0.605736 -2.251896

1 0 -6.576552 0.207191 1.276332

1 0 -3.017656 0.821802 -1.693063

1 0 -7.659436 -1.204733 -0.417600

1 0 -4.213693 -0.600580 -3.302721

16 0 -5.908660 -1.620017 -1.976834

6 0 3.823110 -0.155533 1.378930

6 0 6.009967 0.219924 -0.446308

6 0 4.520446 0.605736 2.251896

6 0 6.594802 0.969576 0.515669

1 0 3.017656 -0.821802 1.693063

1 0 6.576552 -0.207191 -1.276332

1 0 4.213693 0.600580 3.302721

1 0 7.659436 1.204733 0.417600

16 0 5.908660 1.620017 1.976834

MP2/BSS-A

Adduct **7**: Me_4_Se_4_Br_5_^–^

Symmetry *C*_2h_

energy MP2 = –17822.296377 au

Standard orientation

35 0 -1.729795 7.992878 0.000000

34 0 -1.241067 5.217919 0.000000

35 0 -0.742280 2.799745 0.000000

35 0 0.000000 0.000000 0.000000

35 0 0.742280 -2.799745 0.000000

34 0 1.241067 -5.217919 0.000000

35 0 1.729795 -7.992878 0.000000

6 0 0.000000 5.541215 1.462424

1 0 0.233822 6.612016 1.455254

1 0 -0.527441 5.258116 2.383081

1 0 0.873351 4.894993 1.308334

6 0 0.000000 5.541215 -1.462424

1 0 0.873351 4.894993 -1.308334

1 0 -0.527441 5.258116 -2.383081

1 0 0.233822 6.612016 -1.455254

6 0 0.000000 -5.541215 1.462424

1 0 -0.233822 -6.612016 1.455254

1 0 0.527441 -5.258116 2.383081

1 0 -0.873351 -4.894993 1.308334

6 0 0.000000 -5.541215 -1.462424

1 0 -0.873351 -4.894993 -1.308334

1 0 0.527441 -5.258116 -2.383081

1 0 -0.233822 -6.612016 -1.455254

MP2/BSS-A

Adduct **8**: C_8_H_8_Se_4_Br_5_^–^

Symmetry *C*_2h_

energy MP2 = –22771.699403 au

Standard orientation

35 0 -7.512541 -3.120633 0.000000

35 0 -2.599800 -1.074898 0.000000

34 0 -4.975385 -2.086821 0.000000

34 0 -4.889509 -5.720607 0.000000

6 0 -4.488163 -3.217022 1.447612

6 0 -4.488163 -4.566712 1.434029

1 0 -4.176574 -5.100141 2.337465

1 0 -4.142187 -2.651744 2.316912

6 0 -4.488163 -3.217022 -1.447612

6 0 -4.488163 -4.566712 -1.434029

1 0 -4.176574 -5.100141 -2.337465

1 0 -4.142187 -2.651744 -2.316912

35 0 0.000000 0.000000 0.000000

35 0 7.512541 3.120633 0.000000

35 0 2.599800 1.074898 0.000000

34 0 4.975385 2.086821 0.000000

34 0 4.889509 5.720607 0.000000

6 0 4.488163 3.217022 1.447612

6 0 4.488163 4.566712 1.434029

1 0 4.176574 5.100141 2.337465

1 0 4.142187 2.651744 2.316912

6 0 4.488163 3.217022 -1.447612

6 0 4.488163 4.566712 -1.434029

1 0 4.176574 5.100141 -2.337465

1 0 4.142187 2.651744 -2.316912

MP2/BSS-A

Adduct **3**: C_12_H_8_Se_2_Br_2_

Symmetry *C*_s_

energy MP2 = –10405.874832 au

Standard orientation

6 0 1.409422 -1.468355 2.674847

6 0 1.005315 -0.832798 1.478280

6 0 -0.175964 -0.072743 1.505550

6 0 -0.940093 0.060343 2.680996

6 0 -0.502774 -0.548204 3.862194

6 0 0.675756 -1.315324 3.856428

1 0 2.308588 -2.089420 2.668374

1 0 -1.870948 0.631018 2.664763

1 0 -1.086612 -0.433783 4.777005

1 0 1.016135 -1.809178 4.768573

34 0 -0.868696 0.891127 0.000000

34 0 2.180110 -1.032878 0.000000

6 0 1.005315 -0.832798 -1.478280

6 0 1.409422 -1.468355 -2.674847

6 0 0.675756 -1.315324 -3.856428

6 0 -0.502774 -0.548204 -3.862194

6 0 -0.940093 0.060343 -2.680996

6 0 -0.175964 -0.072743 -1.505550

1 0 2.308588 -2.089420 -2.668374

1 0 1.016135 -1.809178 -4.768573

1 0 -1.086612 -0.433783 -4.777005

1 0 -1.870948 0.631018 -2.664763

35 0 1.121622 2.420662 0.000000

35 0 -2.921118 -0.639312 0.000000

MP2/BSS-A

Adduct **4**: C_12_H_8_Se_2_Br_4_

Symmetry *C*_s_

energy MP2 = –15551.139094 au

Standard orientation

6 0 -0.656674 0.646003 2.658205

6 0 -0.585277 -0.127636 1.478779

6 0 0.151988 -1.322719 1.501173

6 0 0.806328 -1.751680 2.671436

6 0 0.699608 -0.989949 3.841079

6 0 -0.030732 0.211121 3.832609

1 0 -1.201017 1.593063 2.647857

1 0 1.399409 -2.668241 2.661044

1 0 1.196920 -1.329329 4.751260

1 0 -0.103083 0.819271 4.736095

34 0 0.322455 -2.508457 0.000000

34 0 -1.591658 0.519611 0.000000

6 0 -0.585277 -0.127636 -1.478779

6 0 -0.656674 0.646003 -2.658205

6 0 -0.030732 0.211121 -3.832609

6 0 0.699608 -0.989949 -3.841079

6 0 0.806328 -1.751680 -2.671436

6 0 0.151988 -1.322719 -1.501173

1 0 -1.201017 1.593063 -2.647857

1 0 -0.103083 0.819271 -4.736095

1 0 1.196920 -1.329329 -4.751260

1 0 1.399409 -2.668241 -2.661044

35 0 -2.162999 -2.922823 0.000000

35 0 2.836186 -2.117516 0.000000

35 0 -0.369788 3.049482 0.000000

35 0 0.723617 5.156843 0.000000
